# Avian-origin influenza A viruses tolerate elevated pyrexic temperatures in mammals

**DOI:** 10.1126/science.adq4691

**Published:** 2025-11-27

**Authors:** Matthew L. Turnbull, Yingxue Wang, Simon Clare, Gauthier Lieber, Stephanie L. Williams, Marko Noerenberg, Akira J. T. Alexander, Sara Clohisey Hendry, Douglas G. Stewart, Joseph Hughes, Simon Swingler, Spyros Lytras, Emma L. Davies, Katherine Harcourt, Katherine Smollett, Rute M. Pinto, Hui-Min Lee, Eleanor R. Gaunt, Colin Loney, Johanna S. Jung, Paul A. Lyons, Darrell R. Kapczynski, Edward Hutchinson, Ana da Silva Filipe, Jeffery K. Taubenberger, Suzannah J. Rihn, J. Kenneth Baillie, Ervin Fodor, Alfredo Castello, Kenneth G. C. Smith, Paul Digard, Sam J. Wilson

**Affiliations:** 1 MRC– University of Glasgow Centre for Virus Research, University of Glasgow, Glasgow, UK; 2 Cambridge Institute of Therapeutic Immunology and Infectious Disease (CITIID), Department of Medicine, University of Cambridge, Cambridge, UK; 3 Sir William Dunn School of Pathology, University of Oxford, Oxford, UK; 4 Viral Pathogenesis and Evolution Section, Laboratory of Infectious Diseases, National Institute of Allergy and Infectious Diseases, National Institutes of Health, Bethesda, MD, USA; 5 Roslin Institute, University of Edinburgh, Edinburgh, UK; 6 Division of Systems Virology, Department of Microbiology and Immunology, The Institute of Medical Science, The University of Tokyo, Tokyo, Japan; 7 US National Poultry Research Center, Athens, GA, USA; 8 The Walter and Eliza Hall Institute of Medical Research (WEHI), Parkville, VIC, Australia; 9 Department of Medical Biology, University of Melbourne, Melbourne, VIC, Australia

## Abstract

Host body temperature can define a virus’s replicative profile—influenza A viruses (IAVs) adapted to 40° to 42°C in birds are less temperature sensitive in vitro compared with human isolates adapted to 33° to 37°C. In this work, we show that avian-origin PB1 polymerase subunits enable IAV replication at elevated temperatures, including avian-origin PB1s from the 1918, 1957, and 1968 pandemic viruses. Using a model system to ensure biosafety, we show that a small increase in body temperature protects against severe disease in mice and that this protection is overcome by a febrile temperature–resistant PB1. These findings indicate that although elevated temperature itself can be a potent antiviral defense, it may not be effective against all influenza strains. These data inform both the clinical use of antipyretics and IAV surveillance efforts.

Fever is an evolutionarily conserved innate immune response and is a common clinical sign of many infections, including influenza in humans (1–3). Influenza A virus (IAV) has two distinct sites of infection in humans—the upper and lower respiratory tract (URT and LRT, respectively). Human-adapted H1N1 and H3N2 seasonal influenza strains preferentially bind to α2,6-linked sialic acid, which is more prevalent on epithelial cells of the URT (4, 5), whereas avian IAVs preferentially target α2,3-linked sialic acid, which is more abundant on cells in the LRT (6–8). LRT involvement is more commonly associated with severe disease, and notoriously pathogenic IAVs of recent history, such as zoonotic H5N1 IAVs and the 1918 H1N1 pandemic virus, both targeted the LRT and caused tissue damage at this site (6–11). Notably, the temperature at which avian IAVs replicate in their natural host (40° to 42°C) is considerably higher than that found in both the URT of humans (33°C), the site at which human-adapted seasonal IAVs pre-dominantly replicate, and the LRT of humans (37°C) (12–17) (Fig. 1A). Accordingly, avian IAVs are reported to be less temperature sensitive than human IAVs at febrile temperatures in cultured human cells (18–26). However, how this influences virus replication and pathogenicity within febrile hosts has not been elucidated. We hypothesized that elevated temperature is a key anti-IAV defense and that the viral genetic determinants that permit replication of avian IAVs at 40° to 42°C would increase disease severity in a febrile mammalian host relative to a temperature-sensitive human seasonal virus. We thus sought to elucidate the viral determinants of temperature-resistant replication and test this in an in vivo mouse model of simulated fever.

## Febrile temperature restricts human, but not avian, IAVs

**Fig. 1:**
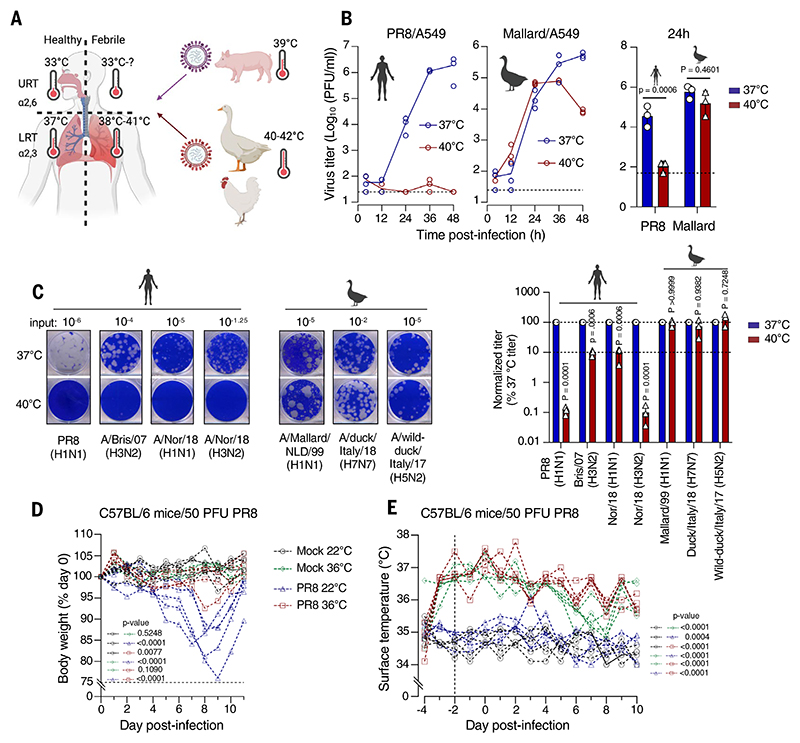
Febrile temperature inhibits IAV in a strain-dependent manner. (**A**) A schematic of temperatures in the human respiratory tract and IAV reservoirs, highlighting the predominant N-linkage of sialic acid receptors in human airways. [Figure created with BioRender.com] (**B**) Infectious yields (quantified through plaque assay on MDCK cells) at the indicated time points from A549 cells, inoculated with a multiplicity of infection (MOI) of 0.001 plaque-forming units (PFU) per cell and cultured at 37°C or 40°C (lines connect the mean titer at each time point from an experiment performed in technical triplicate). Also presented (right) are three independent infectious yield experiments from 24 hpi. (**C**) Normalized titers of the indicated viruses, quantified through plaque assay on MDCK cells, from three independent titrations (means ± SDs) at the indicated temperature. An example dilution is shown for each virus. (**D**) Weight-loss curves for each mouse from groups of five C57BL/6 mice acclimatized to each temperature and infected (or mock infected) with 50 PFU of PR8. (**E**) Surface temperatures for each mouse in (D) (dashed line indicates the end of the acclimatization period). The *P* values were calculated comparing 37°C and 40°C [(A) and (B)] or the indicated comparison using area-under-the-curve analysis [(D) and (E)] using an ordinary two-way analysis of variance (ANOVA) with Tukey’s multiple comparisons test.

We measured virus replication in human lung cells of exemplar human and avian IAVs. A laboratory-adapted human-origin H1N1 IAV [A/Puerto Rico/8/1934 (H1N1) or “PR8”], was heavily attenuated at febrile temperature (40°C) relative to normal core temperature (37°C) in A549 cells, an adenocarcinomic human alveolar cell line (Fig. 1B). By contrast, an avian-origin IAV [A/Mallard/Netherlands/10-Cam/1999 (H1N1) or “Mallard”] replicated efficiently at both temperatures. This effect was mirrored in plaque reduction assays in Madin-Darby canine kidney cells (MDCK cells), where a broader panel of human seasonal IAV isolates (H1N1 and H3N2) were all inhibited by febrile temperatures. Conversely, diverse avian IAVs (H1N1, H5N2, and H7N7) were largely unperturbed by elevated temperature (Fig. 1C and fig. S1A), in accordance with previous observations (18–26). Notably, a representative virus from the 2009 H1N1 swine flu pandemic (pdm2009)—a reassortant with genome segments derived from IAVs infecting humans, pigs, and birds (27)—had an intermediate temperature sensitivity phenotype (fig. S1, B and C). Together, these observations suggest that the core temperature of the prehuman reservoir species influences the restriction imposed by febrile temperature.

## Simulated fever protects against severe infection in vivo

Mice do not mount a classic febrile response to IAV infection (28, 29). However, elevating the ambient temperature beyond 34°C can sustainably increase core body temperature to fever-range hyperthermia (30–33), which we refer to as simulated fever. Thus, it is possible to switch febrile temperature on and off artificially without using a pyrogenic effector, such as lipopolysaccharide (LPS), or an antipyretic drug, such as sodium salicylate, which may have confounding effects on IAV replication by regulating proinflammatory cytokine production (34, 35). All of the C57BL/6 mice infected with a low dose of PR8 and housed at 22°C experienced severe weight loss (Fig. 1D). Conversely, those housed at 36°C, simulating fever, maintained a healthy body weight throughout the infection, as reported previously (36). Mice housed at 36°C had body temperatures at least 2°C higher than those housed at 22°C during the early stages of infection (Fig. 1E). Mouse surface temperatures are a reliable proxy for changes in core temperature (37) and are slightly cooler than core body temperature. This suggests that temperatures equivalent to human fever were achieved in the mice housed at 36°C, in line with fever-range whole-body hyperthermia protocols described by others (30–33, 36). Simulated fever protected against severe IAV-induced weight loss, which shows that hyperthermia can be a major anti-IAV defense in vivo.

## PB1 governs polymerase temperature sensitivity and resistance

**Fig. 2:**
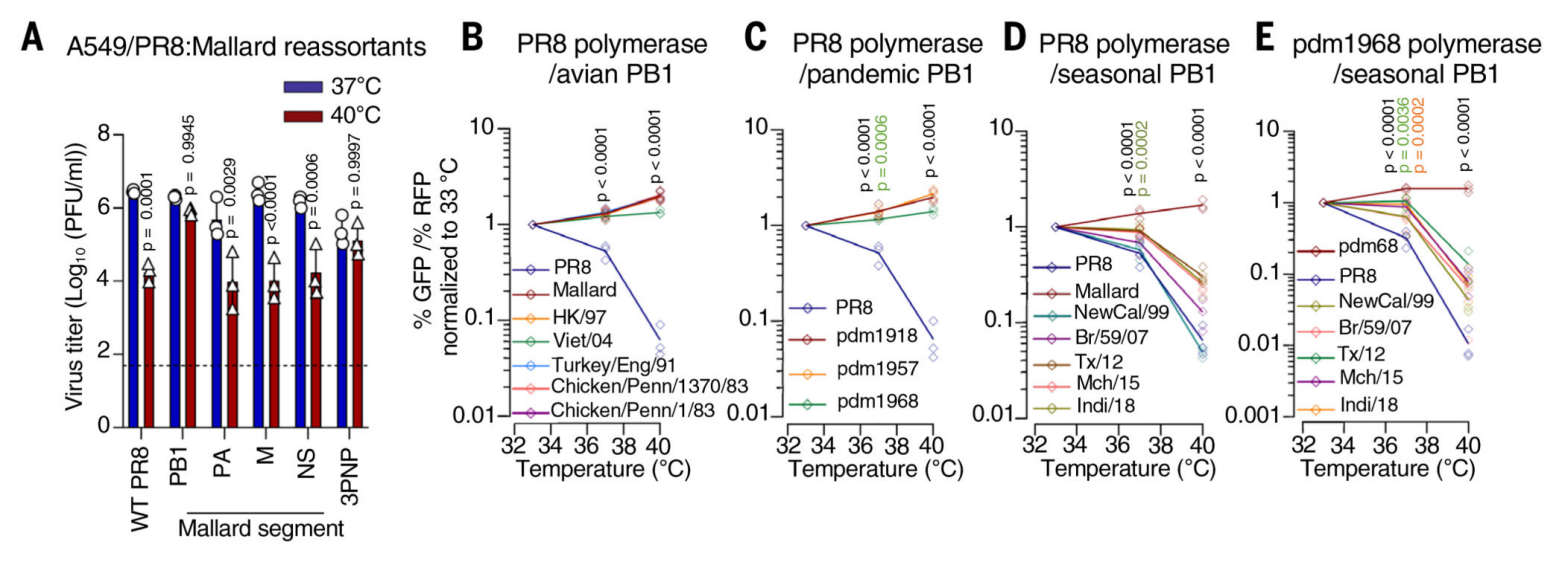
Avian-origin PB1s facilitate temperature-resistant replication in vitro. (**A**) The infectious yield (determined by plaque assay on MDCK cells) from A549 cells infected at an MOI of 0.5 PFU/cell for 15 hours for the indicated PR8-based viruses at either 37°C or 40°C. Data are plotted from three independent experiments. 3PNP refers to PR8 with Mallard polymerase segments (PB2, PB1, PA, and NP: 3PNP). The indicated *P* values were calculated using an ordinary two-way ANOVA with Tukey’s multiple comparisons test comparing yield at 37°C and 40°C. (**B** to **E**) IAV polymerase activity at different temperatures in transfected 293T cells plotted as the proportion of GFP+ cells (viral polymerase) normalized to RFP+ cells (transfection control) for a panel of avian PB1s (B), the three avian-origin pandemic PB1s (C), or a panel of human-adapted seasonal PB1s (D), all using a PR8 polymerase background. Polymerase activity of human-adapted seasonal PB1s in a pdm1968 polymerase background is also shown (E). Data [(B) to (E)] are plotted from three independent experiments executed in triplicate. The indicated *P* values were calculated using an ordinary two-way ANOVA with Tukey’s multiple comparisons test comparing avian-origin PB1s with PR8 PB1 or comparing seasonal PB1s with Mallard (D) or pdm1968 (E) PB1s.

To establish the viral determinants of temperature-resistant IAV replication, we rescued reassortants between the avian-origin and PR8 H1N1 viruses and assessed virus replication in human cells. Notably, the work described in this study was risk assessed and reviewed by a UK biosafety committee and, in the US, by the National Institutes of Health (NIH) Dual Use Research of Concern Institutional Review Entity. The UK Health and Safety Executive was notified about this work (see Biosafety section, below). Only the full polymerase (“3PNP”; containing the Mallard PB2, PB1, PA, and NP segments) and PB1 reassortant viruses replicated robustly at 40°C, which suggests that the PB1 segment is the dominant factor (Fig. 2A and fig. S2, A and B). Replication kinetics and plaque formation of PR8 with a Mallard PB1 segment were similar at 37°C and 40°C, whereas the PR8 parental virus was heavily attenuated (fig. S2, C and D), which confirms the importance of PB1.

The PB1 protein forms part of the heterotrimeric polymerase, which is responsible for the transcription and replication of viral RNA (vRNA) (38). Therefore, we used a polymerase activity assay (39) in which the readout is green fluorescent protein (GFP) fluorescence driven by the viral polymerase normalized to a red fluorescent protein (RFP) transfection control (fig. S2, E and F) to assess the effect of temperature on reassortant polymerases. When the polymerase subunits of the PR8 strain were reconstituted in human cells, polymerase activity was potently inhibited by febrile temperature, an effect that was entirely rescued when the PB1 gene was replaced with that of the Mallard H1N1 virus (fig. S2, G and H). This thermophilic property was retained when the expressions of PB1 segment accessory proteins [PB1-F2 (40) and PB1-N40 (41)] were disrupted individually or together (fig. S2I), which suggests that the full-length PB1 protein is the sensitivity or resistance determinant. Thus, the substantial block caused at 40°C is at the level of polymerase activity and can be entirely rescued by an avian-origin PB1.

## Avian-origin PB1 confers temperature resistance

Next, we performed polymerase activity assays using human IAV polymerases in which the PB1 was replaced by diverse avian PB1s. These included representatives of the 1997 H5N1 (“HK/97”) and the 2003/2004 H5N1 (“Viet/04”) lineage of HPAI H5N1, which caused high mortality in humans (50 to 60%) (42, 43). Notably, these avian PB1s conferred temperature resistance to the PR8 polymerase (Fig. 2B and fig. S3A) and the pdm2009 H1N1 polymerase (fig. S3B). The most severe pandemics of recent history, pdm1918 H1N1 (estimated 20 to 50 million deaths), pdm1957 H2N2 (1 to 4 million deaths), and pdm1968 H3N2 (1 to 4 million deaths), all acquired their PB1 segment, through genetic reassortment, from an avian IAV (44–48). We therefore hypothesized that the PB1 from pdm1918, pdm1957, and pdm1968 viruses would facilitate temperature-resistant polymerase activity. In fact, the avian-origin pandemic PB1s permitted efficient polymerase activity at all temperatures tested (Fig. 2C and fig. S3C).

The 1918, 1957, and 1968 pandemic IAVs circulated in humans for years and eventually became the seasonal influenza outbreaks associated with lower total mortality (typically 290,000 to 650,000 deaths annually) (49). Because these pandemic strains harbored a PB1 that contributed to the polymerase temperature-resistant phenotype, we therefore considered whether adaptive substitutions acquired in PB1 over several decades in human hosts might confer temperature sensitivity to the polymerases of seasonal IAVs. As well as failing to rescue temperature sensitivity of the PR8 and pdm2009 H1N1 polymerases (Fig. 2D and fig. S3, D and E), a panel of human-adapted PB1 genes all ablated the temperature resistance of the 1968 H3N2 pandemic (pdm1968) polymerase (Fig. 2E and fig. S3F). Notably, PR8:PB1 single-gene reassortant viruses showed temperature sensitivity analogous to that of their counterpart polymerase activity assays, with elevated temperature reducing the ability to form plaques between approximately fivefold and ~500-fold (fig. S3G).

The importance of PB1 in determining temperature sensitivity or resistance was confirmed in the context of the PR8 and pdm2009 polymerases, in which the individual subunits of the temperature-resistant pdm1968 polymerase were substituted (fig. S3, H and I). We observed one instance where, like PB1, the pdm1968 PB2 also conferred temperature resistance to the pdm2009 polymerase (fig. S3I). However, this is not a widespread property of PB2, as assessment of a panel of PB2 genes (encompassing 627K and 627E PB2s; table S1) indicated that avian-origin PB2s did not rescue the temperature sensitivity of PR8 (and generally conferred poor polymerase activity at all temperatures; fig. S3J). Moreover, PB2s from human seasonal IAVs did not confer temperature sensitivity to the pdm1968 polymerase (fig. S3K). Thus, polymerase temperature sensitivity appears to be strongly dependent on PB1, although other subunits can contribute in certain contexts.

## Specific PB1 residues enable temperature resistance

**Fig. 3:**
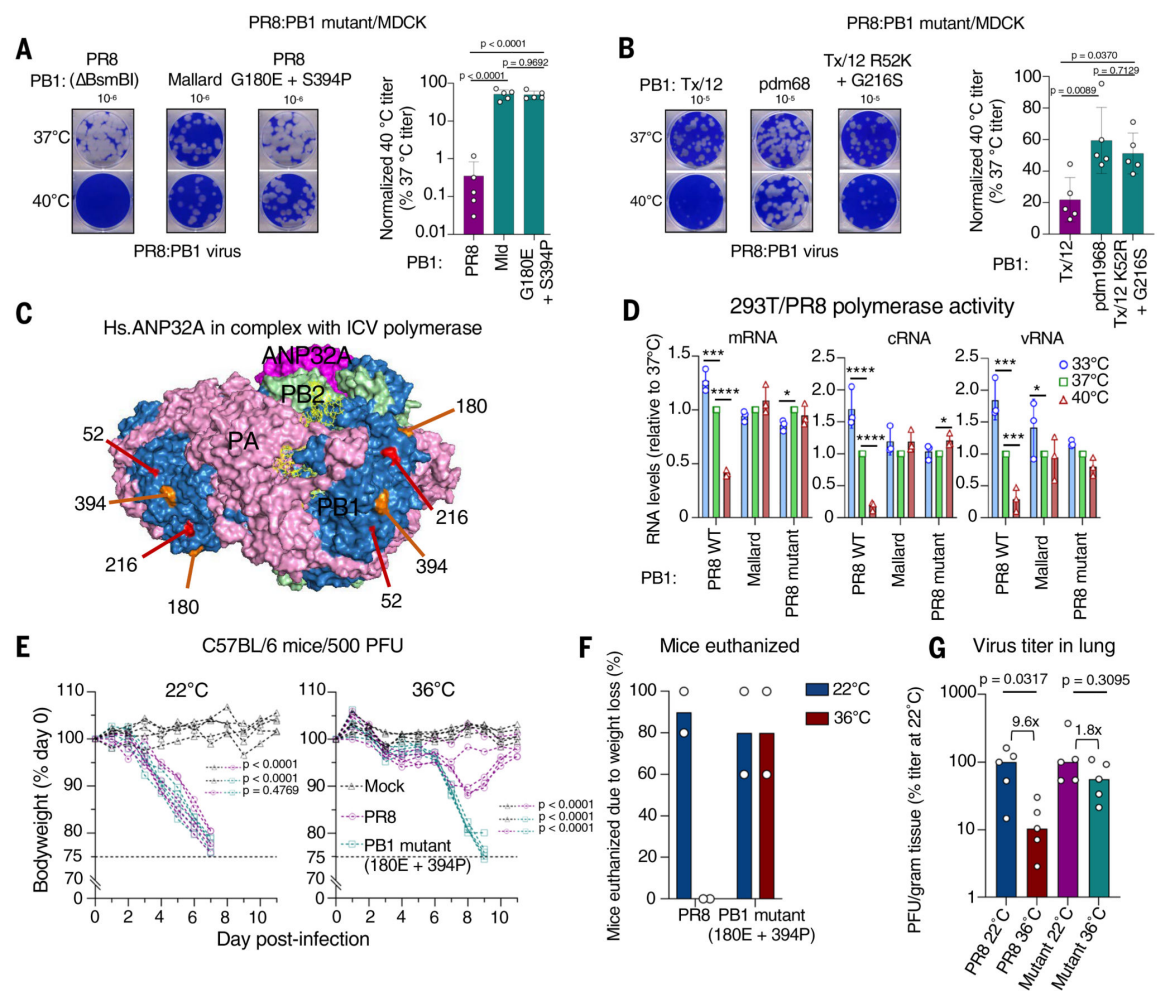
A virus with temperature-resistant mutations in PB1 causes severe weight loss in mice with simulated fever. (**A** and **B**) Normalized 40°C titers (five independent titrations) of the indicated PR8 PB1 reassortants are plotted (± SDs; *P* values from ordinary one-way ANOVA with Tukey’s multiple comparisons tests). Example dilutions are displayed. (**C**) PB1 mutants [from (A) and (B)] are displayed on a model of ANP32A complexed with the viral polymerase (C/Johannesburg/1/66 polymerase, PDB 6XZQ). (**D**) The relative mRNA, vRNA, and cRNA levels (primer extension) from PR8 polymerases containing the indicated PB1 are plotted from three independent experiments. Significant *P* values from two-way ANOVA with Dunnett’s multiple comparisons tests are shown (*P < 0.05; ***P < 0.0006; ****P < 0.0001). (**E**) Weight-loss curves for each mouse from groups of five C57BL/6 mice acclimatized to each temperature and infected with 500 PFU of PR8 or the PB1 mutant (180E + 394P). The dashed line represents the ethical cut-off point for euthanasia. *P* values are from ordinary one-way ANOVA with Tukey’s multiple comparisons tests performed on area-under-the-curve data. (**F**) The proportion of mice euthanized is plotted from (E) combined with an independent repeat experiment. (**G**) Median infectious titer from lung homogenates harvested 24 hpi [infected as in (E) and quantified as in (A)] is plotted from five mice per group. Fold changes and *P* values (two-tailed Mann-Whitney tests) are indicated. Single-letter abbreviations for the amino acid residues are as follows: E, Glu; G, Gly; K, Lys; P, Pro; R, Arg; and S, Ser.

To uncover the specific PB1 residues that confer temperature resistance, we made a series of chimeric PB1 genes based on the PR8 and Mallard variants, which differ at 19 residues (fig. S4A). We screened these chimeric PB1 genes in polymerase assays (fig. S4B), and although it was apparent that multiple configurations of PB1 substitutions could alter the temperature sensitivity of the polymerase, we identified a PR8 PB1 that enabled efficient polymerase activity at 40°C, which contained four substitutions corresponding to the Mallard H1N1 virus (chimera 13; fig. S4B). Mutagenesis of this chimera revealed that two substitutions in PR8 [Gly^180^→Glu (G180E) and S394P] were sufficient to recapitulate most of the temperature-resistant phenotype (Fig. 3A and fig. S5, A and B). We then explored the important residues of the pdm1968 PB1 that confer temperature-resistant replication by making chimeric genes with Tx/12, a direct descendent of pdm1968 that has circulated in humans for decades and renders the pdm1968 polymerase temperature sensitive (Fig. 2E and fig. S3F). The PB1 of Tx/12 differs at 17 residues from pdm1968 PB1 (fig. S4A). As with the PR8-Mallard PB1 chimeras, multiple configurations of PB1 substitutions altered the temperature sensitivity of the pdm68 polymerase, so we selected the chimeric Tx/12 PB1 gene that enabled the most efficient polymerase activity at 40°C with the fewest residues from the pdm1968 PB1 (chimera 14; fig. S4C). Mutagenesis revealed that both substitutions in chimera 14 (R52K and G216S) were required to recapitulate most of the pdm1968 temperature-resistant phenotype (Fig. 3B and fig. S5C). Notably, both the Mallard-PR8 and pdm1968-Tx/12 pairs of substitutions described above reduced temperature sensitivity of a replication-competent virus to levels comparable to the whole PB1 segment from which they were derived (Fig. 3, A and B).

Because the PB1 residues identified in Fig. 3, A and B, were widely separated in the primary protein sequence, we mapped the residues onto a published model of an influenza virus polymerase. The four sites all lay in a line on a surface-exposed region of the polymerase spanning 40.1 Å (Fig. 3C), which suggests that this area of the polymerase is an important determinant of temperature sensitivity or resistance. Sites 52 and 216 are located within the finger subdomain, with site 216 close to the junction between the β-ribbon and finger domain, whereas sites 180 and 394 are in the β-ribbon and β-hairpin subdomains, respectively (50). These sites do not form part of the characterized RNA-dependent RNA polymerase catalytic motifs (51), nor are they proximal to polymerase dimer interfaces (52, 53). To further explore the role of this region of the polymerase in temperature sensitivity or resistance, we examined the ability of the viral polymerase to replicate and transcribe from a vRNA template at a range of temperatures in human 293T cells. The PR8 polymerase was unable to replicate a vRNA template efficiently at an elevated temperature, with both positive sense [complementary RNA (cRNA)] and negative sense genomic (vRNA) replication products substantially reduced (Fig. 3D and fig. S6). We also observed a reduction in mRNA levels, although this may be a secondary consequence of defective genome replication by the polymerase. This phenotype was rescued by both an avian PB1 or the double mutant PB1 (180E + 394P), which permitted efficient production of these RNAs at 40°C (Fig. 3D and fig. S6).

We next analyzed these specific PB1 sites across available IAV sequences. Although the PB1 substitutions at sites 180 and 394 may be atypical (possibly resulting from a laboratory adaptation of the PR8 strain), an analysis of currently circulating subtypes indicated that the majority (88.4%) of avian strains had the K52 and S216 pair, whereas this pairing was not generally favored in humans, occurring in only 1.3% of human H1N1 and 7.1% of human H3N2 PB1s (fig. S7 and table S2). Notably, the K52 and S216 pair was also the predominant configuration for the H2N2 lineage, which circulated in humans for 11 years (1957 to 1968) and was predominant for more than 20 years in the H3N2 lineage (1968 onward), which suggests that the loss of this pairing is not rapidly selected for in humans (fig. S7B).

Furthermore, all the tested PB1s from circulating viruses containing K52 and S216 were temperature resistant in polymerase activity assays (table S3). By contrast, all the temperature-sensitive PB1s had a substitution at one (or both) of these sites. That said, some PB1s, notably those from A/chicken/Pennsylvania/1/1983 (H5N2) and A/chicken/ Pennsylvania/1370/1983 (H5N2), were temperature resistant despite bearing an arginine at position 52, whereas PR8 PB1 conferred temperature sensitivity despite containing K52 and S216 (probably because of positions 180 and 394). Considered together, although having K52 and S216 is a good predictor of thermal resistance, these important residues are not the sole determinant of temperature sensitivity or resistance.

## A PB1 mutant confers fever resistance in vivo

To assess the phenotypic consequences of mammalian infection with a temperature-resistant virus, we compared WT PR8 with the temperature-resistant PB1 mutant (180E + 394P) using C57BL/6 mice with or without simulated fever. In mice housed and infected at 22°C, the wild-type (WT) and mutant viruses caused substantial and nearly identical rates of weight loss, and the majority of mice were humanely euthanized after reaching an ethical end point (Fig. 3, E and F, and fig. S8, A to C). Mice with a simulated fever were protected against WT PR8, showing only moderate weight loss, and none of these mice had to be euthanized because of weight loss. Conversely, the temperature-resistant PB1 mutant caused substantial weight loss despite the simulated fever, and the majority of these mice were humanely euthanized. The protection afforded by simulated fever against the WT virus correlated with an almost 10-fold reduction in viral load at 24 hours postinfection (hpi), whereas the mutant titers were only reduced approximately twofold by simulated fever (Fig. 3G and fig. S8D). Notably, housing mice at elevated temperature did not induce a robust proinflammatory response at this time point (fig. S8E), which indicates that the observed reduction in WT viral load at 24 hpi was the direct effect of elevated temperature on virus replication rather than a boosted host response. Notably, virus titer at 48 hpi was not significantly reduced for either virus (fig. S8F), indicating that, in this model, early virus replication plays a major role in defining disease severity.

Overall, the temperature-resistant replication of the PR8 PB1 mutant characterized in vitro was recapitulated in vivo and overcame the protection mediated by fever-range hyperthermia. These results show that elevated temperature acts as an antiviral mechanism in vivo and that the virus PB1 subunit can overcome this antiviral defense.

## ANP32 origin contextualizes temperature sensitivity

**Fig. 4:**
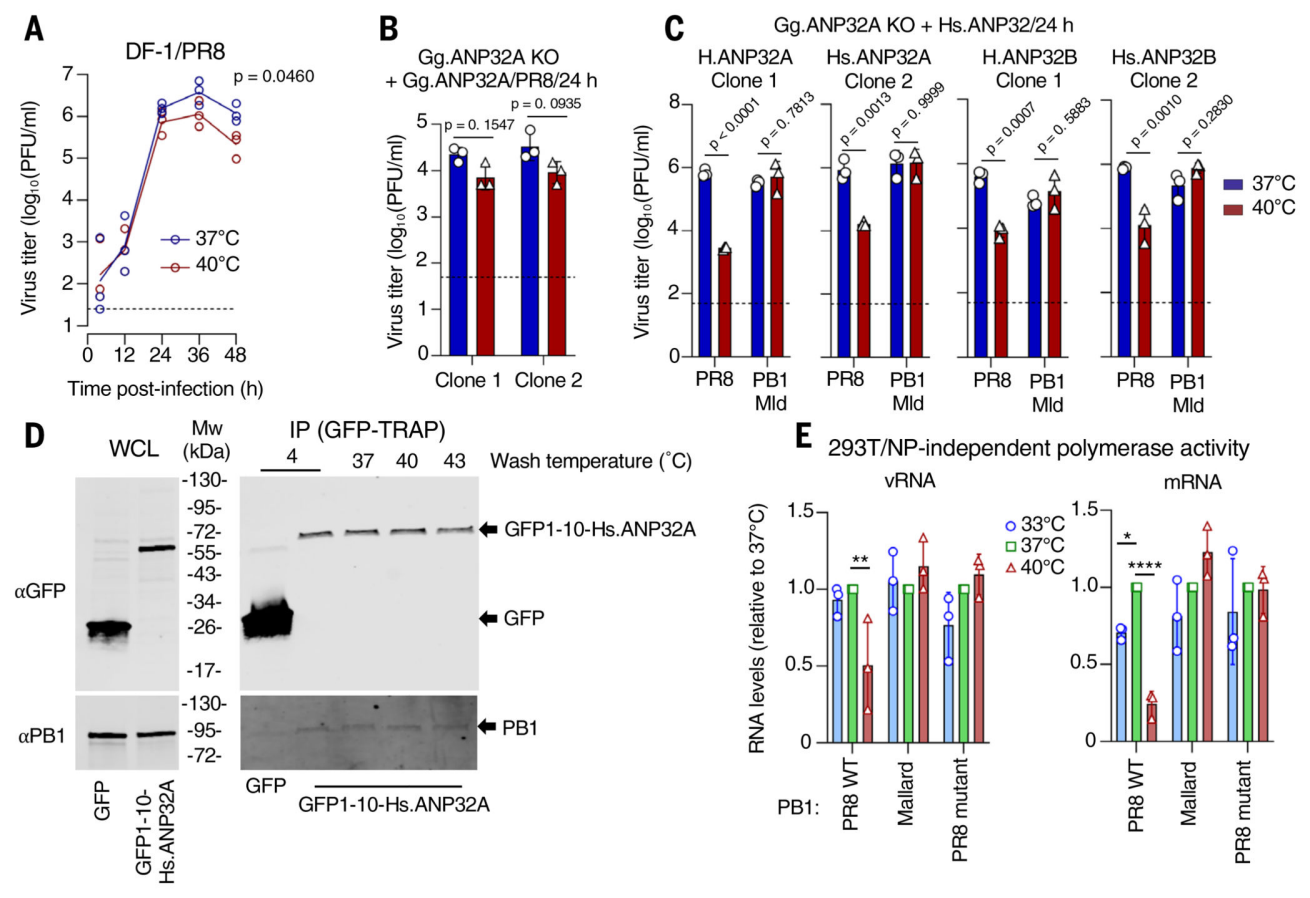
ANP32 species variants contextualize IAV temperature sensitivity. (**A**) Infectious yields at the indicated time points from chicken DF-1 cells inoculated with PR8 (MOI 0.001 PFU/cell) and cultured at 37°C or 40°C (geometric mean from three independent experiments). The dashed line indicates the limit of detection. *P* value was calculated using Welch’s *t* test on area-under-the-curve statistics generated from log-transformed data. (**B**) Infectious yields of PR8 (24 hpi) at the indicated temperature from two DF-1 ANP32A knockout (KO) clones (CRISPR-Cas9), expressing exogenous chicken ANP32A are plotted from three independent experiments. The dashed line indicates the limit of detection. *P* values were calculated using two-way ANOVA with Tukey’s multiple comparisons test on log-transformed data. (**C**) The infectious yields of PR8 and a PR8:Mallard PB1 reassortant in the presence of the indicated human ANP32 are plotted [as in (B)]. (**D**) Western blots of whole-cell lysates (WCLs) or immunoprecipitates (IPs) using GFP-TRAP beads from PR8-infected DF-1 cells (MOI 5 PFU/cell) modified to express GFP-tagged human ANP32A (GFP1-10-Hs.ANP32A) or GFP are shown. Complexes were washed at the indicated temperature before PB1 and ANP32 expression was assessed using Western blotting. (**E**) The relative mRNA and vRNA levels (NP-independent primer extension) from PR8 polymerases containing the indicated PB1 (but no NP) are plotted, as in Fig. 3D.

Waterfowl and poultry are avian influenza reservoirs and, like many birds, have core body temperatures of 40° to 42°C. We investigated whether an IAV that is temperature sensitive in human cells would behave similarly in avian cells. Notably, when DF-1 chicken fibroblasts were infected with PR8 (Fig. 4A) or a pdm2009 H1N1 virus (fig. S9A), virus replication at 40°C was comparable to that at 37°C (although PR8 was still modestly inhibited at 40°C). These findings contrasted with the attenuation observed at febrile temperatures in human cells (Fig. 1B and fig. S1B) and indicated that virus-host interactions affect temperature sensitivity. Because temperature sensitivity mapped to the virus polymerase, we examined host ANP32 proteins (acidic leucine-rich nuclear phosphoprotein 32 kDa), which mediate the assembly of the viral genome replication complex (53) and are required for vRNA and cRNA synthesis during replication (54). In humans, both ANP32A and ANP32B support influenza polymerase activity, whereas in chickens, only ANP32A is required (55–58). We hypothesized that ANP32 affects the temperature sensitivity specified by PB1. To explore this, we used CRISPR-Cas9 to knock out ANP32A in chicken DF-1 cells and then replaced the chicken ANP32A with exogenous human (Hs.) or chicken (Gg.) ANP32s (fig. S9, B to D). We then infected these cells and observed that PR8 was highly temperature sensitive in chicken cells expressing Hs.ANP32A or -B (recapitulating the situation in human cells) but only weakly temperature sensitive when Gg.ANP32A was expressed (Fig. 4, B and C, and fig. S9, E to I). Conversely, the PR8:Mld PB1 virus resisted elevated temperature using either human ANP32 variant (Fig. 4C and fig. S9, J and K). Notably, PR8 replication was robust at febrile temperatures in DF-1 cells expressing both Gg.ANP32A and Hs.ANP32A together, which suggests that human ANP32A is not a dominant-negative factor in these cells (fig. S9L). Overall, this indicates that ANP32 affects IAV temperature sensitivity and that the maximal temperature sensitivity conferred by PB1 is only revealed by a mammalian ANP32 cofactor.

To further test this genetic association, we examined whether we could rescue temperature-resistant replication of PR8 in human cells by supplying exogenous Gg.ANP32A. Expressing exogenous Gg.ANP32A in human cells did not substantially affect replication at 40°C (fig. S10A); therefore, we speculated that Hs.ANP32A and Hs.ANP32B interfere with the Gg.ANP32A-polymerase interaction in human cells. Accordingly, we reduced Hs.ANP32A protein to undetectable levels in human cells using CRISPR-Cas9 and replaced it with exogenous CRISPR-resistant Gg.ANP32A (fig. S10B and table S4). Polymerase activity was enhanced, although not entirely rescued, at 40°C when Gg.ANP32A was expressed (fig. S10C), which suggests that either ANP32 variant could be used in this context. We next ablated Hs.ANP32B in the same lineage to generate human cells only expressing detectable Gg.ANP32A (fig. S10D) and observed that polymerase activity was not substantially attenuated at 40°C when Gg.ANP32A was available (i.e., both copies of Hs.ANP32B were disrupted and three of four copies of Hs.ANP32A were disrupted) (fig. S10E and table S4). Moreover, access to Gg.ANP32A increased PR8 replication at 37°C ~10-fold, and at 40°C, it further boosted PR8 replication in human cells (between ~50- and ~175-fold) relative to parental cells (fig. S10, F to H). As a complement to the boost to replication at higher temperatures in human cells, we observed a substantial rescue of plaque formation in MDCK cells modified to express Gg.ANP32A at 40°C relative to Hs.ANP32A or -B (fig. S10I). However, Gg.ANP32A could not fully reverse the attenuation caused by human febrile temperatures, and virus replication was still reduced at 40°C relative to 37°C. These observations indicate that the species origin of ANP32 contextualizes IAV temperature sensitivity and that maximal temperature sensitivity is only observed with mammalian ANP32. By contrast, Gg.ANP32A can facilitate the replication of a typically temperature-sensitive IAV at febrile temperature (making PB1-dependent temperature-sensitive phenotypes appear modest or absent). Notably, the rescue is typically incomplete and the cellular context also appears important [i.e., other aspect(s) of chicken cells that support temperature-resistant replication and other aspect(s) of human cells that reveal temperature sensitivity]. We speculate that these divergent cellular backgrounds counter the effect of exogenous heterologous ANP32 in the presence of endogenous ANP32, making Gg.ANP32A appear more dominant in chicken cells and Hs.ANP32 appear more dominant in human cells.

One plausible explanation for ANP32 affecting temperature sensitivity could be that human ANP32 proteins do not physically interact with the virus polymerase efficiently at fever-range temperature. Thus, we examined the ability of Hs.ANP32A to remain complexed with the viral polymerase in vitro at elevated temperatures. We used a GFP1-10 tag to mediate pulldown of the ANP32-polymerase complex, which did not perturb the ability of ANP32A to support virus replication (fig. S11, A to C). In cell-free conditions, Hs.ANP32A remained complexed with PB1 from a temperature-sensitive virus (PR8) across a range of temperatures (4° to 43°C), which suggests that ANP32 physically interacts with the viral polymerase in a fashion that is not obviously thermolabile (Fig. 4D). Given that mutation of PR8 PB1 can rescue temperature-sensitive genome replication (Fig. 3D), we considered the key PB1 residues in relation to ANP32 using a model of the ANP32-bound IAV polymerase dimer (53). PB1 sites 180 and 394, as well as sites 52 and 216, were clearly distal to the areas where the leucine-rich repeat (LRR) region of ANP32 interacts with polymerase subunits (Fig. 3C). However, the unresolved C-terminal low-complexity acidic region (LCAR) of ANP32 could potentially interact with PB1 at these sites. Because the LCAR has been proposed to promote the recruitment of NP to nascent RNA (59), we examined polymerase activity in the absence of NP using a 47–nucleotide (nt) template. Notably, polymerase temperature sensitivity was maintained, which suggests that the mechanism of temperature sensitivity is likely NP independent (Fig. 4E and fig. S11, D and E). Because temperature sensitivity is not explained by ANP32 or NP interactions, we speculate that conformational changes in PB1 can be affected at elevated temperature, preventing the ANP32-bound polymerase from adopting conformation(s) required for replicase activity.

## Discussion

How fever benefits the host is often enigmatic. In this work, we reveal that elevated temperature itself likely mediates potent anti-IAV activity in vivo, supporting the notion that the temperature component of fever is an ancient and potent antiviral defense. Notably, avian-origin PB1 enables IAV to resist inhibition at febrile temperatures. Whereas previous studies have highlighted the importance of host origin in determining the temperature sensitivity of IAVs in vitro (18–26), the fever-range hyperthermia model used in this work shows that temperature-sensitive replication in vitro, in the absence of an immune system or the complexity of multiorgan systems, can be reflected in vivo and substantially affect disease severity. A virus with two amino acid substitutions in PB1 that replicated efficiently at an elevated temperature (G180E + S394P) also caused severe disease in the presence of a simulated fever, despite causing essentially identical weight loss to the parental virus in nonfebrile mice. This, alongside our in vitro data, indicates that elevated temperature itself was the principal anti-IAV mechanism in this model rather than a temperature-dependent priming of immune processes (1) or altered gut microbiota–dependent host responses (36).

Notably, avian-origin IAVs, such as H5N1 lineages or pandemic IAVs containing an avian-origin PB1, replicate more efficiently at febrile temperatures and may overcome the protective effect of elevated temperature. This could plausibly explain why the PB1 segment from the highly pathogenic 1918 pandemic virus enables an otherwise mild or seasonal H1N1 virus to colonize the LRT of ferrets (ferret core body temperature is ~38° to 40°C), whereas other segments, including HA and NA, do not substantially alter tissue tropism (60). Notably, cold-adapted IAV vaccine strains also struggle to colonize the LRT of ferrets and mice (61, 62), and this thermal restriction of replication is widely accepted as a major mechanism for the attenuation and safety of these vaccines in humans. Considered together, febrile temperature appears to represent an underappreciated barrier to LRT tropism (and associated disease severity), which can be overcome by IAV PB1.

Accordingly, a major implication of this study is that human studies should be conducted to determine whether the antiviral role of elevated temperature is as potent an antiviral defense in real-world settings, as is implied by laboratory experiments. For example, does elevated temperature protect against seasonal IAVs but not pandemic viruses or avian spillover viruses? Moreover, are poor febrile responses associated with severe seasonal influenza, and could the clinical and domestic use of antipyretics exacerbate pathogenesis and enhance the transmission of seasonal IAVs? Fever is often treated with antipyretic medication (63, 64); however, there is clinical evidence that treating fever may not always be beneficial to the patient [(65–67), reviewed in (68, 69)] and may even promote IAV transmission in humans at the population level (70). Suppression of fever can enhance viral replication in animal models of human-adapted IAVs and other common pathogens (70–73). Furthermore, IAV-infected patients who are afebrile but symptomatic can show higher mortality and require longer hospital stays than febrile patients (74). Moreover, basal temperatures can be lower in elderly patients and their febrile responses blunted relative to younger adults [reviewed in (75)], which potentially contributes directly to the increased mortality observed in elderly patients. Our findings complement these studies and may explain some of the molecular details that underpin them.

In this work, we linked PB1-dependent temperature sensitivity to a genetic interaction with the host factor ANP32, indicating that temperature sensitivity is governed by host and viral components of the replicase. Avian-origin PB1 did not substantially increase basal polymerase activity in human cells at lower temperatures, and a polymerase containing avian PB1 is similarly active at 37°C and 40°C. Thus, febrile temperature resistance would be unlikely to compensate, at any temperature, for the severe attenuation caused by a PB2 that cannot recruit human ANP32. Although adaptation of the viral polymerase to higher temperatures in birds was expected, the ability of avian ANP32A to substantially rescue otherwise temperature-sensitive replication was unanticipated. Given that temperature-resistant PB1 is widespread in avian viruses, temperature-resistant replication is likely beneficial for the virus in birds. PR8 is still weakly temperature sensitive in avian cells (at human febrile temperatures), which suggests that the configuration of PB1 is still important for maximal replication in the avian host. Robust temperature sensitivity of IAV is only revealed with mammalian ANP32s. It is currently unclear whether the contribution of human ANP32A or -B to extreme temperature-sensitive IAV replication has been selected for or has arisen by chance. It is not immediately clear how the PB1 residues 52, 180, 216, and 394 that we linked to temperature sensitivity or resistance might affect the ANP32-containing replicase. However, we note that PB1 residue 180 is located in the dynamic β-ribbon of the replicase structure, and all the residues lying adjacent could plausibly contribute to the large conformational changes required to move the β-finger to open the template entry channel and facilitate replicase activity. We speculate that these conformational changes are affected by elevated temperature in human-adapted PB1s. Notably, substitutions in PB1 adjacent to the residues identified in our study have been reported as important for the temperature sensitivity of the FluMist vaccine (76, 77). These observations support the hypothesis that PB1 plays a leading role in determining temperature sensitivity. However, PB2, PA, and the interplay between the PB2 and PB1 subunits have also been implicated in the temperature sensitivity of the IAV polymerase, as have PB2 residues that facilitate functional cooperation with ANP32 proteins, such as sites 627 and 701 (78–87). Thus, despite the apparent dominance of PB1 in this study, other polymerase subunits apparently contribute to determining temperature sensitivity.

It is unclear why the PB1 proteins from past pandemic viruses lose their temperature resistance in the years after each pandemic. This may be linked to a preference for replication or transmission in the cooler URT of humans. Alternatively, relaxing selection of viruses that replicate efficiently at temperatures above 38°C might lead to temperature-resistant residues being lost as a result of host-specific compositional biases (88, 89) or being selected against in scenarios where lower pathogenicity favors transmission (90).

We acknowledge limitations in our study. For example, the PB1 residues that we identified may represent examples of many possible configurations that govern polymerase temperature sensitivity, and only one atypical configuration has been examined in vivo. Furthermore, although fever-range hyperthermia reveals a protective role for elevated temperature, it does not recapitulate the full pathophysiological innate fever response. Moreover, we cannot be certain of the precise temperatures at which IAV is replicating in the respiratory tract of mice with simulated fever, although we can expect this to be above normal core temperature (30–33, 36). Nonetheless, this work highlights that a small increase in core body temperature equivalent to a mild fever, can be the difference between mild symptoms and severe or lethal disease. The linking of temperature sensitivity in vitro to reduced disease severity in simulated febrile hosts raises the possibility that elevated temperature itself could reduce the severity of many viral diseases (as many viruses are temperature sensitive in cultured cells). In future, consideration of fever-resistant virus replication may help assess the severity of pandemic threats, such as from the highly pathogenic 2.3.4.4b H5N1 lineage (91, 92), and inform the use of antipyretics in the clinic and the home.

## Materials and methods summary

### Ethics statement

All animal experiments were carried out under the authority of a UK Home Office Project License (PP1769824) within the terms and conditions of the strict regulations of the UK Home Office Animals (scientific procedures) Act 1986 and the Code of Practice for the housing and care of animals bred, supplied, or used for scientific purposes. Before Home Office approval, the license was reviewed through the Cambridge University Animal Welfare and Ethics Review Body. Individual studies were checked against the approved animal license internally by senior animal technicians. Mice were euthanized by cervical dislocation when they reached 75% body weight from their starting body weight, in line with the humane end points in the animal license.

### Biosafety

All work in this study involving infectious virus or replicons was conducted in the UK (no infectious work was conducted in the US). In accordance with recommendations by the UK Health and Safety Executive, the work described in this study was risk assessed, reviewed by a biosafety committee, and the Health and Safety Executive was notified about the work (GM activity 223/13.2a, GM223/19.1, and GM353/24.2) and updated with relevant results. The NIH Dual Use Research of Concern (DURC) Institutional Review Entity reviewed this manuscript before publication (IRE-24-M-01). The committee determined that this research did not meet the definition of DURC because “the results, information, and technology are not reasonably anticipated to be directly misapplied to have a significant impact on public health.”

All the viruses used in this study were handled at containment level 2 (CL2). Where reverse genetics was used to make an internal-gene reassortant or a PB1-mutant virus, the well-characterized and laboratory-adapted IAV PR8 was used as a backbone. PR8 has been shown to be avirulent in humans, and this is governed by the PR8 glycoprotein genes (HA and NA) (93, 94). Many IAVs frequently cause a fever in humans, but because PR8 is avirulent (and therefore does not elicit fever), we reasoned that fever is not a key defense against PR8 in humans and that engineering temperature-resistant PR8 reassortants or mutants would be unlikely to increase the infection or disease severity caused by this virus in humans. No changes to the PR8 glycoprotein genes were made in any reverse genetics–derived virus used in this study; thus, the tropism and antigenicity of these viruses were predicted to be the same as with the parental PR8. The temperature-resistant PB1 mutant (G180E + S394P), used in animal experiments, replicated to comparable titers as to the parental PR8 virus in vitro (at 37°C), and thus there was no reason to believe that this virus would be more pathogenic in mice housed under standard laboratory conditions before initiating experiments (PR8 is already highly pathogenic in mice).

### Cells and viruses

Cell lines were cultured under standard conditions in medium supplemented with 9% (v/v) heat-inactivated fetal bovine serum (FBS) and 10 μg/ml gentamicin and the relevant antibiotics used to select cells transduced with lentiviral vectors (95). CRISPR-Cas9–mediated knockout of ANP32 genes (pLentiCRISPRv2) and exogenous expression of ANP32 (p-Δ*Sfi*I-ΔRFP-SCRPSY) were achieved using lentiviral vectors under standard conditions (95), as described previously (96, 97). CRISPR edits were assessed using Oxford Nanopore –based sequencing of polymerase chain reaction (PCR) amplicons targeting the edited region (95).

PR8 [A/Puerto Rico/8/1934 (H1N1)], pdm2009 [A/California/04-061-MA/2009 (H1N1)], Mallard [A/Mallard/Netherlands/10-Cam/1999 (H1N1)], and reassortant viruses were generated using established reverse genetics protocols (95), essentially as described previously (98). Other strains used were progeny of the original isolates (95).

### Virus infection and replicon assays

Infectivity was assessed using serially diluted virus and enumeration by plaque assay using MDCK cells and an Avicel R-591 overlay under standard conditions (95). For replicon assays (95), 293T cells were transfected with plasmids encoding the minimal components of the influenza A polymerase (PB2, PB1, PA, and NP), a so-called minigenome [vRNA-like RNA with a GFP open reading frame (ORF) in place of a viral ORF], and TagRFP (to normalize for transfection efficiency). Fluorescent cells were quantified using flow cytometry and plotted as a proportion of GFP+ cells normalized to RFP+ cells (unless otherwise stated). To assess NP-independent polymerase activity, a plasmid encoded 47-nt vRNA-like template (based on the NP segment) was used under standard conditions (95) and was quantified using a primer extension assay, as previously described (99).

### Immunoprecipitation and Western blotting

GFP1-10–tagged ANP32 was immunoprecipitated using GFP–telomeric repeat amplification protocol (TRAP) agarose beads (Proteintech, GTA-20) under standard conditions (95). Protein expression was assessed using standard Western blotting approaches (95). Briefly, sonicated cell lysates were resolved using acrylamide gels (Life Technologies) before wet transfer onto a suitable membrane and probing with the relevant validated primary antibodies (95). Bands were visualized using DyLight-labeled secondary antibodies (Thermo Fisher Scientific) before scanning with a Li-Cor Odyssey imaging system (95).

### Mouse infections

Female 6-week-old C57BL/6 mice (Charles River) were housed in individual ventilated cages under standard conditions or placed inside an incubator (Techniplast). The initial incubator temperature was 30°C, and this was increased in increments of 1°C per day for 6 days to reach a final temperature of 36°C for the remainder of the experiment. Non–heat-treated mice remained at an ambient temperature of 22°C throughout the experiment (95). On day 8 of heat treatment, groups of five mice housed at 36°C or 22°C were intranasally infected with PR8 or the temperature-resistant PB1 mutant (180E + 394P) (95). Mouse body weights and body temperatures were measured daily (95). Lung homogenates were prepared for assessment of infectious titer (plaque assay on MDCK cells) and analysis of cytokines and chemokines by enzyme-linked immunosorbent assay (ELISA) (R&D Systems) using standard approaches (95).

### Residue distribution analysis

Representative PB1 protein sequences were aligned using Clustal Omega, the alignment was used for maximum likelihood phylogenetic analysis using standard approaches (95), and the residue counts at the sites of interest were extracted from subtrees from each pandemic lineage (1918, 1957, 1968, and 2009). Alternatively, human, avian, or swine PB1 and PB2 protein sequences were aligned separately by host group using mafft. The distribution of residues for each site of interest (PB1 sites 52, 216, 180, and 394 and PB2 site 627) was calculated from the alignments and visualized using WebLogo or plotly (95).

## Supplementary Material

science.org/doi/10.1126/science.adq4691

Materials and Methods; Figs. S1 to S11; Tables S1 to S6; MDAR Reproducibility Checklist

Supplementary Material

## Data Availability

All data are available in the manuscript, supplementary materials, or the underlying data (100). Experimental materials generated in this study are available through a materials transfer agreement with the University of Edinburgh or the University of Cambridge.
