# Supplementary material for: Avian-origin influenza A viruses tolerate elevated pyrexic temperatures in mammals
Source: Science. Author manuscript; Available in PMC 2026 Jan 7. (PMC7618609; doi:10.1126/science.adq4691)
Supplement: Supplementary Material [file EMS211202-supplement-Supplementary_Material.zip › science.adq4691_sm.pdf]

Science
AAAS

Supplementary Materials for

Avian-origin influenza A viruses tolerate elevated pyrexic temperatures in mammals

Matthew L. Turnbull *et al*.

Corresponding author: Sam J. Wilson, sjw58@cam.ac.uk

Science **390**, eadq4691 (2025)
DOI: 10.1126/science.adq4691

The PDF file includes:

Materials and Methods
Figs. S1 to S11
Tables S1 to S6

Other Supplementary Material for this manuscript includes the following:

MDAR Reproducibility Checklist

Materials and Methods

Cells and viruses.

Madin Darby Canine Kidney (MDCK; RRID:CVCL_0422) cells , human embryonic kidney 293T cells (293T; RRID:CVCL_0063), human adenocarcinomic alveolar basal epithelial (A549; RRID:CVCL_0023) cells and chicken fibroblasts (DF-1; RRID:CVCL_0570) were grown in Dulbecco's Modified Eagle Medium (DMEM) supplemented with 9% (v/v) heat-inactivated fetal bovine serum (FBS) and 10 µg/ml gentamicin. Sialyltransferase 1 overexpressing MDCK cells (MDCK-SIAT1; RRID: CVCL_Z936) were cultured as above with the addition of 1 mg/ml geneticin. A549 and 293T cells were authenticated using short tandem repeat (STR) analysis. The species origin of MDCK and DF-1 cells was confirmed by sequencing Mx1 and ANP32A cDNAs respectively.

A/Puerto Rico/8/1934 (H1N1) ('PR8'; kind gift of R. Fouchier; GenBank accessions: PB2 EF467818, PB1 EF467819, PA EF467820, HA EF467821, NP EF467822, NA EF467823, M EF467824, NS EF467817), A/California/04-061-MA/2009 (H1N1) ('pdm2009'; kind gift of D. Perez; GenBank accessions: PB2 KX134889.1, PB1 KX136823.1, PA KX134973.1, HA KX136363.1, NP KX136508.1, NA KX135184.1, M KX136570.1, and NS KX134783.1) and A/Mallard/Netherlands/10-Cam/1999 (H1N1) ('mallard'; kind gift of L. Tiley; GenBank accessions: PB2 KC209512.1, PB1 KC209513.1, PA KC209514.1, HA KC209515.1, NP: KC209516.1, NA KC209517.1, M KC209518.1, NS KC209519.1) viruses were generated by reverse genetics essentially as described previously (98). Briefly, 10^6 human 293T cells were transfected with pHW2000 reverse genetics plasmids (250 ng plasmid for each virus segment), using 8 µl of 1 mg/ml PEI. For 7:1 PB1 reassortants, the PB1 gene segments described in this study (see Table S5) were chemically synthesized with flanking directional *Bsm*BI sites in pUC57-

AmpR (GeneWiz) and the *Bsm*BI fragment was sub-cloned into the pHW2000 reverse genetics vector. On day 2, the media was changed to serum-free DMEM supplemented with 0.14 % Bovine Serum Albumin (w/v) and 1 µg/ml L-(tosylamido-2-phenyl) ethyl chloromethyl ketone (TPCK)-treated trypsin ('Virus growth medium'). Virus-containing supernatant collected on day 4 was used to inoculate flasks of MDCK cells maintained in virus growth medium. Clarified supernatant was collected when approximately 90% of cells demonstrated cytopathic effect (typically 36 – 48 h post-infection). Virus was titrated by standard plaque assay on MDCK cells.

Human seasonal IAV A/Brisbane/10/2007 (H3N2), a kind gift of Professor Alan Hay (World Influenza Centre, London) was cultured in MDCK-SIAT1 cells, A/Norway/3433/2018 (H1N1) (GISAID accession EPI_ISL_391292) and A/Norway/3275/2018 (H3N2) (GISAID accession EPI_ISL_390020) were propagated on MDCK cells and were a kind gift of Professor John McCauley (Francis Crick Institute, London). A/duck/Italy/18VIR4932-2/2018 (H7N7) and A/wild-duck/Italy/17VIR6926-1/2017 (H5N2) were a gift from Isabelle Monne (Istituto Zooprofilattico Sperimentale delle Venezie (IZSVe)) and isolated in embryonated chicken eggs for one passage and then propagated on MDCK-Gg.ANP32A cells. All H5N1 subtype avian/1918 and 1957 pandemic PB1 or PB2 genes used in polymerase assays were exclusively the ORF of PB1 or PB2 (no UTR sequence of the segment was included for biosafety reasons) and the pcDNA-PB1/2 was sequence-verified to be 100% identity at the protein level to the isolate sequence published on GenBank (Tables S1 and S5). We attempted to clone more PB2 genes (i.e. all the counterparts of the PB1 genes described in Table S5) but found the sequences highly unstable, thus we were limited to the genes described in Table S1.

Chimeric PB1 genes were made using overlap extension PCR (See Table S6 for overlapping oligos and templates) flanked with directional *Bsm*BI sites (forward oligo: 5'- gca acc

cgt ctc tgg gga gcg aaa gca ggc aaa cca ttt g-3' and reverse oligo 5'-ggt tgc cgt ctc tta tta gta gaa aca agg cat ttt ttc atg aag g-3') to sub-clone the *Bsm*BI fragment into the reverse genetics pHW2000 vector.

Site-directed mutagenesis was employed to generate IAV point mutants using the Agilent QuikChange Lightning Site-directed Mutagenesis kit according to manufacturer's instructions. For PR8 PB1 mutants, the two existing *Bsm*BI sites were first ablated with silent changes in PB1 (a591g and c1287g according to GenBank accession numbering for EF467819) to allow *Bsm*BI-directed sub-cloning into the pHW2000 vector using the following pairs of oligos: a591 (5'- tca tat tgt ctc tca ccc gcc tct ttc tct gaa aat gag -3' and 5'- ctc att ttc aga gaa aga ggc ggg tga gag aca ata tga -3') and c1287g (5' – aag att cag gat gga cac gcc taa tac agt gct taa c -3' and 5'- gtt aag cac tgt att agg cgt gtc cat cct gaa tct t -3'). For other PB1 mutants, the following pairs of oligos were used: PR8 PB1 G180E (5'-tct ttc tct gaa aat gag ttg tga ttt cca ttt ctt ctt tgt tca ttg act c-3' and 5'-gag tca atg aac aaa gaa gaa atg gaa atc aca act cat ttt cag aga aag a-3'), PR8 PB1 S394P (5'-gaa aga aga ttg aaa aaa tcc gac ctc tct taa tag agg gga ctg cat c-3' and 5'- gat gca gtc ccc tct att aag aga ggt cgg att ttt tca atc ttc ttt c-3'), PR8 PB1 chimera 13 D175N (5' gtg ttg tta ttt cca ttt ctt ctt tgt tca ttg att cca tca cat ctt tga gg-3' and 5'- cct caa aga tgt gat gga atc aat gaa caa aga aga aat gga aat aac aac ac-3'), PR8 PB1 chimera 13 E180G (5'- ctc ttt ctc tgg aaa tgt gtt gtt atc ccc att tct tct tta tcc att gat tcc -3' and 5'- gga atc aat gga taa aga aga aat ggg gat aac aac aca ttt cca gag aaa gag-3'), PR8 PB1 chimera 13 P394S (5'-ggc tgt gcc atc tat tag cag cga tcg gat ttt ttc aat ctt ctt-3' and 5'- aag aag att gaa aaa atc cga tcg ctg cta ata gat ggc aca gcc-3'), PR8 PB1 chimera 13 D398E (5'-gag gct gtg ccc tct att agc aga ggt cgg att-3' and 5'- aat ccg acc tct gct aat aga ggg cac agc ctc -3'), Tx/12 PB1 R52K (5'-gtc gtc cac ttc cct ttc tct gaa tat tgg tgt gtt ctg ttg a-3' and 5'-tca aca gaa cac acc aat att cag aga aag gga agt gga cga c-3'), Tx/12 G216S (5'- gga aga aaa agc aaa gag tga ata

aga gaa gct acc taa taa gag-3’ and 5’- ctc tta tta ggt agc ttc tct tat tca ctc ttt gct ttt tct tcc-3’), Mallard PB1 ΔN40 (PB1 M40V; atg > gtg) (5’- tct gtt gac tgt gtc cac ggt gta tcc tgt tcc tg -3’ and 5’- cag gaa cag gat aca ccg tgg aca cag tca aca ga -3’) and Mallard PB1 ΔF2 (silent cat>cac at codon 32 of PB1: 5’- tgt tcc tgt tcc gtg gct gta tgg agg atc tcc -3’ and 5’- gga gat cct cca tac agc cac gga aca gga aca -3’).

To make cells constitutively expressing ANP32 proteins, the corresponding sequences of human ANP32A (GenBank accession: NM_006305.3), chicken ANP32A with 33 amino acid insertion (GenBank accession: XM_413932.5), human ANP32B (GenBank:NM_006401.2) and chicken ANP32B (GenBank accession: NM_001030934.1) ORFs or their CRISPR-resistant derivatives (see below) were chemically synthesized with flanking directional *Sfi*I sites in pUC57-AmpR (GeneWiz) and the *Sfi*I fragment was sub-cloned into the lentiviral transfer vector plasmids pΔ*Sfi*I-SCRPSY or p-Δ*Sfi*I-ΔRFP-SCRPSY, which have been described previously (96). Lentiviral vectors were made by cotransfecting 293T cells with pNLGP, pΔ*Sfi*I-SCRPSY-ANP32 or p-Δ*Sfi*I-ΔRFP-SCRPSY-ANP32 and pVSV-G at a ratio of 5:5:1 as described previously (97). 0.22 µm-filtered lentiviral vector-containing cell supernatant was used to transduce mammalian cells which were selected using 2 µg/ml puromycin (or 10 µg/ml for MDCK cells).

To make cells expressing N-terminally GFP1-10 tagged ANP32A, first the pcDNA3.1(+) plasmid was modified to include directional *Sfi*I sites in the MCS by annealing and ligating a pair of oligos (5’- tcg ata *ggc cga gag ggc c*at ccc ggg ta*g gcc tct ctg gcc*-3’ and 5’-tcg a*gg cca gag agg cc*t acc cgg gat *ggc cct ctc ggc c*ta-3’) into *Xho*I-digested pcDNA3.1(+). Next, a GFP1-10-linker-Hs.ANP32A fragment with 5’ *Not*I and 3’ *Xho*I sites was created by overlap extension PCR using pQCXIP-GFP1-10 (kind gift from Prof. Olivier Schwartz) and p-Δ*Sfi*I-ΔRFP-SCRPSY-CRISPR-R-Hs.ANP32A as templates (oligos: 5’-aag ctt ggt acc ggc cga gag ggc cgc ggc cgc acc atg gtg

agc aag ggc gag gag ctg ttc -3', 5'- cac cgc ctc cgg atc cac cgc ctc cct tct cgt tgg ggt ctt tgc tca gg-3', 5'- cgg tgg atc cgg agg cgg tgg atc aat gga gat ggg cag acg gat tca ttt ag-3', and 5'- tga tac tct aga ggc cag aga ggc cct cga gtt agt cat cat ctt ctc cct cat ctt c-3'). The stop codon of GFP1-10 was removed and an in-frame flexible glycine-serine linker (GGGGSGGGGS) was included, with unique *Kpn*2I and *Bam*HI sites, and sub-cloned into the pcDNA3.1(+)-*Sfi*I plasmid. The *Sfi*I fragment was then sub-cloned into the lentiviral vector plasmid p-Δ*Sfi*I-ΔRFP-SCRPSY. A lentiviral vector was then made to transduce cells as described above.

Virus infections.

Cells were washed with serum-free DMEM prior to inoculation with virus diluted in serum-free DMEM to achieve the required MOI. After a 1 h adsorption step at 37°C, cells were overlaid with serum-free DMEM supplemented with 0.14 % BSA fraction V for single-cycle assays, with the addition of 1 μg/ml TPCK-treated trypsin for multicycle- and plaque assays, and then incubated at the required temperature. For plaque assays, MDCK cells were typically plated in 6-well plates to achieve an even monolayer on the day of infection. Cells were infected with serially diluted virus as described above, with the addition of Avicel R-591 to a final concentration of 1.2% in the overlay medium. To titrate infectious virus in the supernatant of infected cells, plaque assays were incubated at the permissive temperature of 37°C. For temperature-specific plaque reduction assays, cells were adsorbed at 37°C for 1 h and then shifted to the desired temperature immediately after overlay. To visualize plaques by immunostaining, formaldehyde-fixed MDCK cells were permeabilized with PBS/0.2% (v/v) Triton-X100 and immunostained for viral NP using mouse monoclonal anti-IAV NP (Bio-Rad Cat# MCA400, RRID:AB_2151884) and goat anti-mouse IgG (H + L)-HRP conjugate (Bio-Rad Cat# 1721011, RRID:AB_2617113), then visualized with KPL TrueBlue Peroxidase Substrate (5510-0030, Insight Biotechnology).

GFP reporter polymerase activity assays.

To express the components of the IAV polymerase in transfected 293T cells, the following plasmids were employed: reverse genetics plasmid pHW2000 for PR8 and pdm2009 polymerase genes, reverse genetics plasmid pLLB for Pdm1968 polymerase genes (kind gift from Earl Brown, University of Ottawa, Canada. GenBank accessions: PB2 KY321924.1, PB1 KY321925.1, PA KY321926.1, NP KY321928.1), or protein-expression pcDNA3.1 for pandemic or H5 avian PB1 genes. Importantly, the same class (reverse genetics versus protein expression plasmids) of plasmid was used for comparison of each PB1 gene within an experiment. 293T cells seeded in 96-well plates were transfected in triplicate with 8.3 ng each of polymerase gene expressing plasmid to reconstitute the minimal components of the influenza polymerase (PB2, PB1, PA, NP), 100 ng of an RNA polymerase I promoter-containing plasmid to express a vRNA-like RNA with the GFP ORF in place of a viral gene ORF, and 10 ng of pCMV-TagRFP-C (Evrogen) to constitutively express TagRFP to normalize for transfection efficiency, using PEI in serum-free medium. Cells were incubated in opti-MEM (Gibco) at the desired temperature after the addition of transfection mix for 48 h. The cells were then dispersed with trypsin and fixed prior to flow cytometry on a MerckMilliPore GUAVA HT benchtop flow cytometer. Flow cytometry data were analyzed using FlowJo. A No-PB1 control was included to set up gating to remove background levels of signal. Data are plotted as a proportion of GFP+ cells normalized to RFP+ cells unless otherwise stated, for example some plots are plotted as activity relative to 33°C following subtraction of the mean background (No PB1) at each temperature.

Transfection for vRNP reconstitution and NP-independent replication.

293T cells were seeded in 12-well plates 24 hours before transfection at approximately $6.0x10^5$ cells/well to achieve 70-80% confluency at 37°C. Expression pcDNA3.1 plasmids containing the coding regions for PR8 polymerase genes PB2, PB1 and PA were transfected at 500 ng. The PR8 NP-encoding plasmid was transfected at 1000 ng. A pPolI plasmid encoding the WSN-NA influenza vRNA was transfected at 500 ng. For NP-independent assays, 293T cells were seeded in 24-well plates 24 hours before transfection to achieve 70-80% confluency at 37°C and 100 ng of the pcDNA PR8-PB2, -PA and mutant PB1 plasmids were transfected (omitting the pcDNA PR8-NP plasmid). 50 ng of a pPolI plasmid encoding a 47nt vRNA-like template based on the NP segment was cotransfected using Lipofectamine 2000 (Thermo Fisher Scientific Inc.) in accordance with the manufacturer's protocols. Transfected plates were incubated at either 33-, 37- or 40°C in parallel for 24 hours. At 24 hours post-transfection, 500 µl of TRI Reagent® (Sigma) was added, and total RNA was extracted following the manufacturer's protocols for subsequent primer extension assay as previously described (99).

RNA extraction and primer extension assay.

TRI Reagent® (Sigma) was used to isolate total RNA following the manufacturer's protocols. First-strand RNA synthesis via SuperScript III (Thermo Fisher Scientific Inc.) was conducted using NA-specific or NP-specific [γ-^{32}P] ATP (PerkinElmer) radiolabeled primers. A primer specific to 5S rRNA was used as an internal control. Products were resolved on a 6% or 12% denaturing PAGE gel with 7 M urea. The resulting gels were imaged by phosphorimaging through a FLA-5000 scanner (Fuji), and targets were quantified using Fiji ImageJ2. Experiments were done in triplicate.

Expression of PB1 and NP in transfections for primer extension assays.

Cells were lysed using Promega lysis buffer (Promega). Factory protocols were followed for subsequent western blotting. Primary antibodies for anti-PB1 (GeneTex Cat# GTX125923, RRID: AB_2753122) and anti-NP (GeneTex Cat# GTX125989, RRID: AB_11168364) from GeneTex were used. The primary anti-β-actin antibody was supplied by Santa Cruz Biotechnology (Santa Cruz Biotechnology Cat# sc-47778, RRID: AB_626632). Secondary, conjugated HRP goat anti-rabbit IgG (Li-COR 926-32211) and anti-mouse IgG (Li-COR 926-32220) antibodies were used before detection with the Li-COR Odyssey DLx platform. Images were processed with the affiliated software (LI-COR, Inc, 2022).

CRISPR-Cas9 modification to ablate ANP32 protein expression in clonal cells.

CRISPR-Cas9 mediated knockout of ANP32 genes was achieved using the pLentiCRISPRv2 one vector system following protocols from the Zhang lab. Guides directed against ANP32 genes were designed with a 20 nt target and a 3’ NGG PAM sequence using the CHOPCHOP web-based tool and pairs of oligonucleotides were annealed with directional *Bsm*BI overhangs to subclone into pLentiCRISPRv2 with either a hygromycin B or a blasticidin resistance marker. Lentiviral vectors were made as described above and used to transduce cells which were selected with 200 μg/ml hygromycin B or 5 μg/ml blasticidin. Bulk populations of cells targeted with at least 7 guides and 2 non-targeting control guides were screened by western-blot analysis of ANP32 protein expression. Two bulk cell populations using two separate guides that were positive for evidence of knockout, and the NTC controls, were single-cell cloned by limiting dilution to create at least two stable knockout cells. The following pairs of oligonucleotides were successful and used in this study: for Hs.ANP32A guide 1 (targeting exon 4): 5’-cac cgc cgt cga

gat atg tga gtt g -3’ and 5’- aaa cca act cac ata tct cga cgg c-3’, for Hs.ANP32A guide 2 (targeting exon 3): 5’-cac cgg gtc cga atg act tac cag -3’ and 5’- aaa cct ggt aag tca ttc gga ccc-3’, Gg.ANP32A guide 1 (targeting exon 3): 5’- cac cgc atc ccg atc gta gcc atc g-3’ and 5’- aaa ccg atg gct acg atc ggg atg c-3’, and Gg.ANP32A guide 2 (targeting exon 5): 5’-cac cga gag gag gac gta agc gga g-3’ and 5’-aaa cct ccg ctt acg tcc tcc tct c-3’, Hs.ANP32B guide 1 (targeting exon 4): 5’-cac cgc ttg gat ggc tat gac cga g -3’ and 5’-aaa cct cgg tca tag cca tcc aag c -3’ and Hs.ANP32B guide 2 (targeting exon 2): 5’-cac cga cag gtt cga gaa ctt gtc t -3’ and 5’-aaa cag aca agt tct cga acc tgt c -3’, and NTC guide: 5’-cac cgt gac gta ccg ctg gag gta -3’ and 5’- aaa cta cct cca gcg gta cgt cac-3’.

To rescue expression of Hs.ANP32A or Gg.ANP32A in CRISPR-Cas9 modified cell lines, a CRISPR-resistant version of each gene was designed by making silent mutations in regions of high homology with successful guides against both the human and chicken ANP32 sequences. The following changes were introduced (nucleotide numbering begins at adenine of start codon): for CRISPR-resistant Hs.ANP32A (a99g, c102g, a105g, c111g, a114c, c270g, g273c, t276c, a279g, t282c, t283c, a285g, t288g, a429c, t432c, c435g, t444c, g450c, c456t, t537c, t540c, a543g, t546c, t549c, a597g, t612c, a615c) and for CRISPR-resistant Gg.ANP32A (a423g, c426g, a429c, c435g, t438c, t447c, g450c, t453c, a459g, a462g, a693c, a696g, a708g, a714c). The corresponding DNA sequence was synthesized in pUC57-Amp (Bio Basic) with flanking *Sfi*I sites and the *Sfi*I fragment was sub-cloned into p-Δ*Sfi*I-ΔRFP-SCRPSY prior to lentiviral vector production and cell transduction as described above. The cells were infected early after selection to mitigate for any undesired CRISPR-Cas9 activity and/or decay in expression over passage.

Protein expression analysis and antibodies.

To generate cell lysates, 1-2 x 10^6 adhered cells were washed with PBS and lysed in 500 µl SDS sample buffer (12.5% glycerol, 175 mM Tris-HCl [pH 8.5], 2.5% SDS, 70 mM 2-mercaptoethanol, 0.5% bromophenol blue). Proteins were subsequently separated by SDS-PAGE on NuPage 4% to 12% Bis-Tris polyacrylamide gels and transferred onto nitrocellulose membranes. Immunoblotted membranes were subsequently probed with a mouse monoclonal anti-actin (JLA20 hybridoma; courtesy of the Developmental Studies Hybridoma Bank, University of Iowa; (DSHB Cat# jla20, RRID: AB_528068) or a mouse monoclonal anti-GAPDH (Proteintech Cat# 60004-1-Ig, RRID: AB_2107436) acting as a loading control. To probe for anti-actin, lysates were diluted 1:10 and run in parallel to avoid oversaturation. For ANP32 proteins, the following antibodies were employed: rabbit polyclonal anti-ANP32A (Abcam Cat# ab51013, RRID: AB_881992) for human ANP32A, rabbit polyclonal anti-PHAPI (LSBio, LS-B10851) for human and chicken ANP32A, and rabbit monoclonal anti-Hs.ANP32B (Abcam, ab184565) for human and chicken ANP32B (which also cross-reacts with ANP32A)**.** To detect the GFP component of GFP1-10-ANP32A in functional validation assays, a rabbit monoclonal anti-GFP (Cell Signaling Technology Cat# 2956, RRID: AB_1196615) were used. Thereafter, membranes were probed with species-specific DyLight-labelled goat secondary antibodies (Thermo) and scanned using a LiCor Odyssey scanner.

Immunoprecipitation of GFP1-10-ANP32A with PB1.

To generate cell lysates, 1.04 x 10^7 DF-1-ANP32A-KO cells overexpressing GFP1-10-ANP32A, or DF-1-eGFP cells serving as a control, were infected with PR8 virus at an MOI of 5 (PFU/cell) for 8 h. Cells were washed with ice-cold PBS and then lysed in 2.5 ml RIPA buffer (50mM Tris pH 7.5, 150 mM NaCl, 0.1% Sodium dodecyl sulfate (SDS), 0.5% sodium deoxycholate, 1% Triton X 100) containing 5 mM DTT and 50 µg/ml AEBSF protease inhibitor

at 4°C. Lysates were precleared by centrifugation for 5 minutes at 16000 x *g* at 4°C. 500 µl lysate per condition were incubated with 20 µl GFP-TRAP agarose beads (Proteintech, GTA-20) equilibrated in lysis buffer containing 2 µg/µl Benzonase (Millipore, #70746-4) for 16 h at 4°C with gentle agitation. Beads were washed twice with 1 ml ice cold lysis buffer. Beads were washed 3 times with 1ml prewarmed D-PBS at the indicated temperatures for 2 minutes (4°C, 37°C, 40°C, 43°C). A final wash step was performed with ice cold lysis buffer. Proteins were eluted in 40 µl 1.5x LDS loading buffer containing 5mM DTT at 95°C. Proteins were separated on Biorad 4–20% Mini-PROTEAN® TGX™ and transferred onto nitrocellulose membranes. Membranes were probed with rat monoclonal anti-GFP (Proteintech, 3h9-150) and rabbit polyclonal anti-influenza A virus PB1 (Thermo Fisher Scientific Cat# PA5-34914, RRID: AB_2552264). Goat fluorescence conjugated secondary antibodies were used to visualize on a LiCor Odyssey scanner.

Mouse infections.

Female 6-week-old C57BL/6 mice (Charles River 632 C57BL/6J) were housed in individual ventilated cages (IVC) containing nesting material and environmental enrichment at an ambient temperature of 22˚C for seven days to acclimatize into the facility after transport. To house mice at 36˚C, heat treatment groups were placed inside a heated incubator containing racking to accommodate their home IVC cages (Techniplast). The temperature in the incubator was initially set at 30˚C and increased in increments of 1˚C per day for 6 days to reach a final temperature of 36˚C for the remainder of the experiment. The temperature of the cage was monitored using a digital thermometer connected to a probe placed inside the sealed cage. Non-heat-treated mice remained at an ambient temperature of 22˚C throughout the experiment.

On day 8 of heat treatment, groups of 5 mice housed at 36˚C or 22˚C were infected (or mock infected) with PR8 or the 'temperature-resistant' PB1 mutant (180E + 394P) prepared in an inoculum volume of 50 µl sterile DPBS (Sigma). To perform intranasal infections, mice were lightly anesthetized with isoflurane. As soon as a state of consistent deep breathing was detected the mouse was infected with 50 µl of prepared inoculum via pipetting directly onto the nose, allowing the mouse to inhale the inoculum into the lungs. The mouse was then returned to its home cage. Mouse body weights were recorded daily from all groups. Individual mice were identified via standard ear notching.

Mouse Temperature measurements.

Mouse body temperatures were measured daily using an Avita FT90 Touch Free Infrared Thermometer. Each mouse was placed onto the grid of its home cage, held lightly by the tail and allowed to grip onto the grid with its forelegs whilst its rear legs were lifted slightly to clearly expose its rear end. The temperature reading was taken from its anal region, directly below the tail.

Infectious virus titration in mouse lung tissue.

The whole soft tissue of the lung was removed following cull and immediately frozen. On the day of titration, the blinded lung tissue was thawed and kept on ice prior to being weighed. The tissue was then homogenized in 1 mL of chilled serum-free DMEM containing 10 µg/ml gentamicin using an Omni Tissue Master 125 homogenizer (Camlab). The homogenates were clarified by centrifugation (14500 x *g*, 5 mins, 4˚C) and infectious virus in the clarified supernatant was titrated by plaque assay on MDCK cells prior to unblinding.

Mouse lung cytokine/chemokine ELISA.

Infectious virus in clarified lung homogenate was inactivated by incubation with Triton-X100 (1% v/v) and ELISAs were performed as per manufacturer's instructions for murine IL-6 (R&D Systems, M6000B-1), MIP-1β (R&D Systems, MMB00) and murine TNF- α (R&D Systems, MTA00B).

Identifying CRISPR-Cas9 genome edits using Oxford Nanopore Technology. Genomic DNA was extracted from CRISPR-Cas9 edited single-cell clones using the QIAamp DNA mini kit as instructed by manufacturer (Qiagen). To sequence the CRISPR-Cas9 lesions on genomic DNA, a 750 bp region flanking the CRISPR-Cas9 target site was PCR-amplified using Taq polymerase (Promega) and primers targeting intronic sequences: For ANP32A guide 1: WL-327 G1F ANP32A: 5'-AGTCAATCACTGTTAAGACAGAAGC-3' and WL-328-G1RC-ANP32A 5'-ATTTCCATGAGCATGGGACTCATGC-3', for ANP32B guide 1: WL-337-G1seq-ANP32B: 5'-TATTTCTTTAGACTACTATGCTGCC-3' and WL-332-G1RC-ANP32B: 5'-TCTTCTGAGTAGCTAAGACTCCAGG-3', and for ANP32B guide 2: WL-333-G2F-ANP32B: 5'-ATCTTCTGGGCCTTAAGGTAGACCC-3' and WL-334-G2RC-ANP32B: 5'-TGGGCCTTAGAGAGATTAAGTAACC-3'. Gel purified (Invitrogen PureLink Gel Extraction kit) PCR products were sequenced by Oxford Nanopore Technology as follows: Sequencing libraries were prepared from the amplicons using the Native barcoding kit (ONT, SQK-NBD114.96). First dA-Tail was added using NEBNext Ultra II End Repair and dA-Tailing module (NEB, E7546) followed by ligation of ONT Native barcodes with Blunt/TA ligase master mix (NEB, M0367). Libraries were pooled and the sequencing adapter added using NEBNext Quick T4 DNA ligase (NEB, E6056) and sequenced on a R.10.4.1 flow cell (ONT, FLO-MIN114) MinKnow version 23.04.5 with Guppy version 6.5.7 using Super-accurate basecalling at 400 bps and requiring barcode at both ends for demultiplexing.

Oxford Nanopore reads were trimmed of adapters using Porechop v0.2.4 (https://github.com/rrwick/Porechop) and quality filtered using NanoFilt v2.8.0 using a quality threshold of 12 (https://github.com/wdecoster/nanofilt). Quality filtered reads were aligned to the genomic region flanking each respective target site using minimap2 v2.17-r941 (https://github.com/lh3/minimap2) and converted to BAM format using samtools v1.12 (https://www.htslib.org). The frequency of each base, insertion and deletion at each site and the sequence of the insertions and deletions were extracted using DiversiTools v0.1 (https://github.com/josephhughes/DiversiTools).

Quantification of prevalence of specific PB1 residues in IAV strains.

All PB1 protein sequences were downloaded from NCBI's Influenza Flu Resource (https://www.ncbi.nlm.nih.gov/genomes/FLU/Database/). These protein sequences were clustered at 99% similarity using CD-HIT v4.8.1 (https://sites.google.com/view/cd-hit/home) to reduce the dataset to a single representative sequence from each cluster to save on downstream compute time. The representative protein sequences were aligned using Clustal Omega v1.2.4 and the alignment was used for maximum likelihood phylogenetic analysis using RAxML v1.0.2 (https://cme.h-its.org/exelixis/web/software/raxml/). The phylogeny was visualized in FigTree v1.4.4 (https://github.com/rambaut/figtree) and subtrees corresponding to each of the pandemic lineages (1918, 1957, 1968 and 2009) were pruned. These subtrees were used to sort the sequences belonging to each pandemic lineage into independent alignments. The residue counts at the sites of interest were extracted from each of these alignments.

Residue distribution analysis.

Sequences for the PB1 and PB2 proteins were downloaded from the NCBI Influenza Virus Database as of the 26th of August 2022. Sequences of type A influenza strains with both proteins of interest being available, isolated in human, avian or swine hosts were retrieved along with the associated metadata. It was ensured that strain, serotype and sampling year information were available for all sequences and only one pair of PB1-PB2 sequences was maintained for each unique virus strain name. All PB1 and PB2 protein sequences were aligned separately by host group using mafft v7.453 (--localpair option) (https://mafft.cbrc.jp/alignment/software/). The distribution of residues for each site of interest (PB1 sites 52, 216, 180, 394; and PB2 site 627) was calculated from the alignments using biopython in Python3 (https://biopython.org). Sequences containing ambiguous amino acids (X) in any of the sites of interest were removed from the analysis. This resulted in a total of 7014 avian, 2259 human and 4145 swine isolate sequence pairs. Site distributions were visualized in Fig. S7b using WebLogo (https://weblogo.berkeley.edu/logo.cgi). Chronological distribution of residues in PB1 sites 52 and 216 was visualized using the plotly Python3 package [https://plotly.com/python/] (Fig S7c).

Statistical analysis.

All statistical tests were performed in GraphPad Prism 10 (https://www.graphpad.com/) and the relevant p-values are indicated in the figures. The statistical test and the number of animals or biological repeats for each comparison is listed in the figure legends.

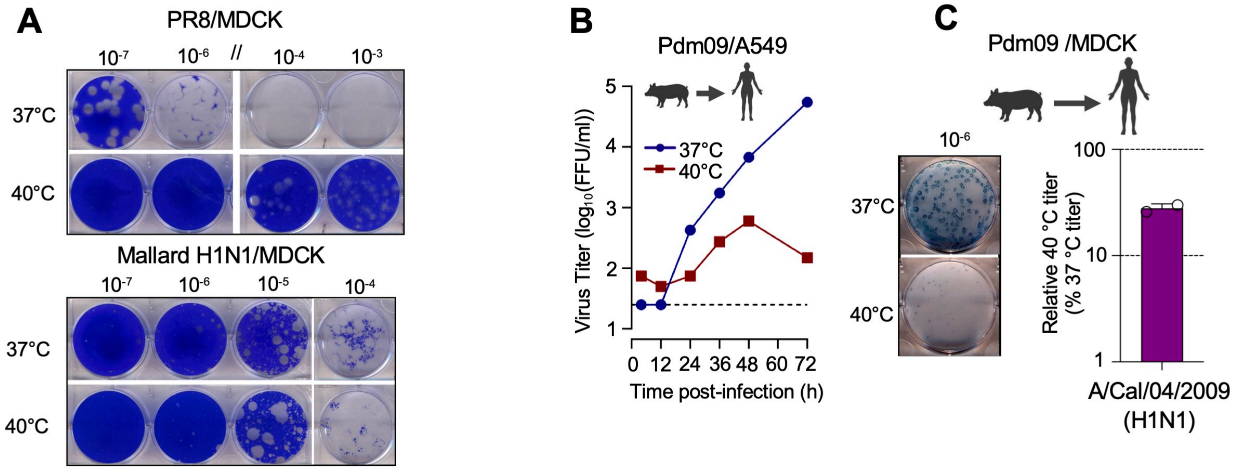

Fig. S1. **Strain-specific inhibition of IAV at febrile temperature *in vitro*. (A)** Example titration of a temperate-sensitive virus (top) and a temperature-resistant virus (bottom) in MDCK plaque reduction assay at 37°C and 40°C. Shown is a more extensive titration of the PR8 and Mallard samples shown in Fig. 1C. **(B)** The infectious yield of a pandemic 2009 H1N1 strain from A549 cells infected at an MOI of 0.001 FFU/cell is plotted. (C) Infectious titer at 40°C (infectious foci in MDCK cells) is plotted as a percentage of the 37°C titer for the pdm09 virus (mean and SD) from two independent titrations.

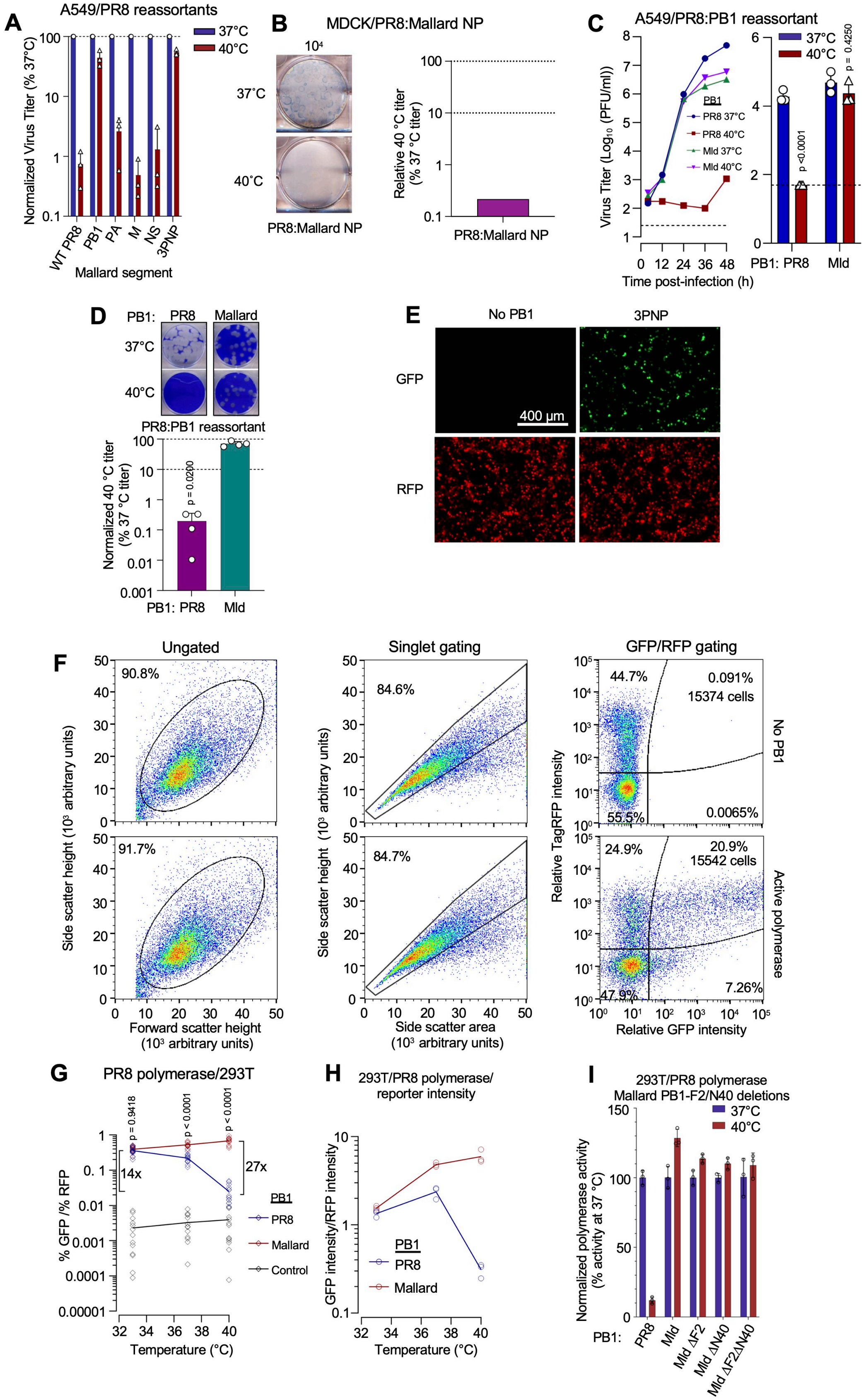
A
A549/PR8 reassortants
Normalized Virus Titer (% 37°C)
37°C
40°C
WT PR8
PB1
PA
M
NS
3PNP
Mallard segment
B
MDCK/PR8:Mallard NP
37°C
40°C
PR8:Mallard NP
Relative 40 °C titer (% 37 °C titer)
C
A549/PR8:PB1 reassortant
Virus Titer (Log10 (PFU/ml))
PB1
PR8 37°C
PR8 40°C
Mld 37°C
Mld 40°C
p <0.0001
p = 0.4250
Time post-infection (h)
PB1: PR8
Mld
D
PB1: PR8 Mallard
PR8:PB1 reassortant
Normalized 40 °C titer (% 37 °C titer)
p = 0.0200
E
No PB1
3PNP
GFP
RFP
400 µm
F
Ungated
Singlet gating
GFP/RFP gating
Side scatter height (10³ arbitrary units)
Forward scatter height (10³ arbitrary units)
Side scatter area (10³ arbitrary units)
Relative TagRFP intensity
Relative GFP intensity
No PB1
Active polymerase
15374 cells
15542 cells
G
PR8 polymerase/293T
% GFP /% RFP
14x
27x
p = 0.9418
p < 0.0001
PR8
Mallard
Control
Temperature (°C)
H
293T/PR8 polymerase/ reporter intensity
GFP intensity/RFP intensity
I
293T/PR8 polymerase Mallard PB1-F2/N40 deletions
Normalized polymerase activity (% activity at 37 °C)
Mld ΔF2
Mld ΔN40
Mld ΔF2ΔN40

Fig. S2. **Mallard PB1 enables temperature-resistant replication in mammalian cells**. **(A)** Normalized titres from the experiment described in Fig. 2A. **(B)** The infectious yield from A549 cells (as in Fig 2A) infected with a PR8: Mallard NP-reassortant virus (that does not plaque in MDCK cells). Infected foci (stained with anti-NP) are shown (left) alongside the infectious yield at 40˚C, plotted as a percentage of the yield at 37˚C (right). (**C**) Infectious yield from A549 cells (infected with MOI 0.001 PFU/cell with the indicated virus) quantified by plaque assay in MDCK cells is shown. Virus was harvested at multiple timepoints (left) or at 24 hours postinfection in three independent experiments (right). Dashed lines denote limit of detection and p-values were calculated as in Fig 1B. (**D**) Infectious titer (plaque assay on MDCK cells) from four independent titrations of the indicated viruses are plotted (mean + SD) as a percentage of the titer at 37˚C. Example wells are also shown (10^{-6} dilution). Data were confirmed to lie on a normal distribution by the Shapiro-Wilk test and p-value is shown for Welch's t-test (**E**) Images of transfected 293T cells visualizing fluorescent protein expression from a GFP-encoding viral minigenome and an RFP transfection control are shown (with a 400 μm scale bar indicated). (**F**) Flow cytometry gating strategy using an example negative control (no PB1, top row) and an active polymerase (bottom row) is shown. Initial gating of 20 000 events using forward and side scatter height intensity is shown (leftmost plot). Single cell populations (singlets) were then gated on side scatter area and side scatter height (middle plot). TagRFP and GFP intensities of single events were then gated into four populations (TagRFP-/GFP-, TagRFP+/GFP-, TagRFP-/GFP+, TagRFP+/GFP+) after compensation to correct for spectral spillover (rightmost plot). The annotated percentage shows the proportion of events that were gated. The total number of cells in the final gating plot is annotated in the top right quadrant. (**G**) PR8 (or PR8 with a Mallard PB1 segment) polymerase activity at different temperatures (as in E and F) is plotted as the mean proportion of GFP+ cells normalized to RFP+ cells (using 13 independent experiments, each performed in technical triplicate, that are described throughout this study). Controls represent a polymerase subunit lacking either PB2 or PB1 (replaced by an empty vector). P-values were calculated as in Fig 2B-E. (**H**) Example polymerase activity assay in 293T cells where relative GFP: RFP intensity is plotted. Data is from three experimental replicates. (**I**) Polymerase activity of a PR8 polymerase harbouring Mallard PB1 protein with indicated PB1-F2 and/or -N40 start codon deletions is plotted from three replicates.

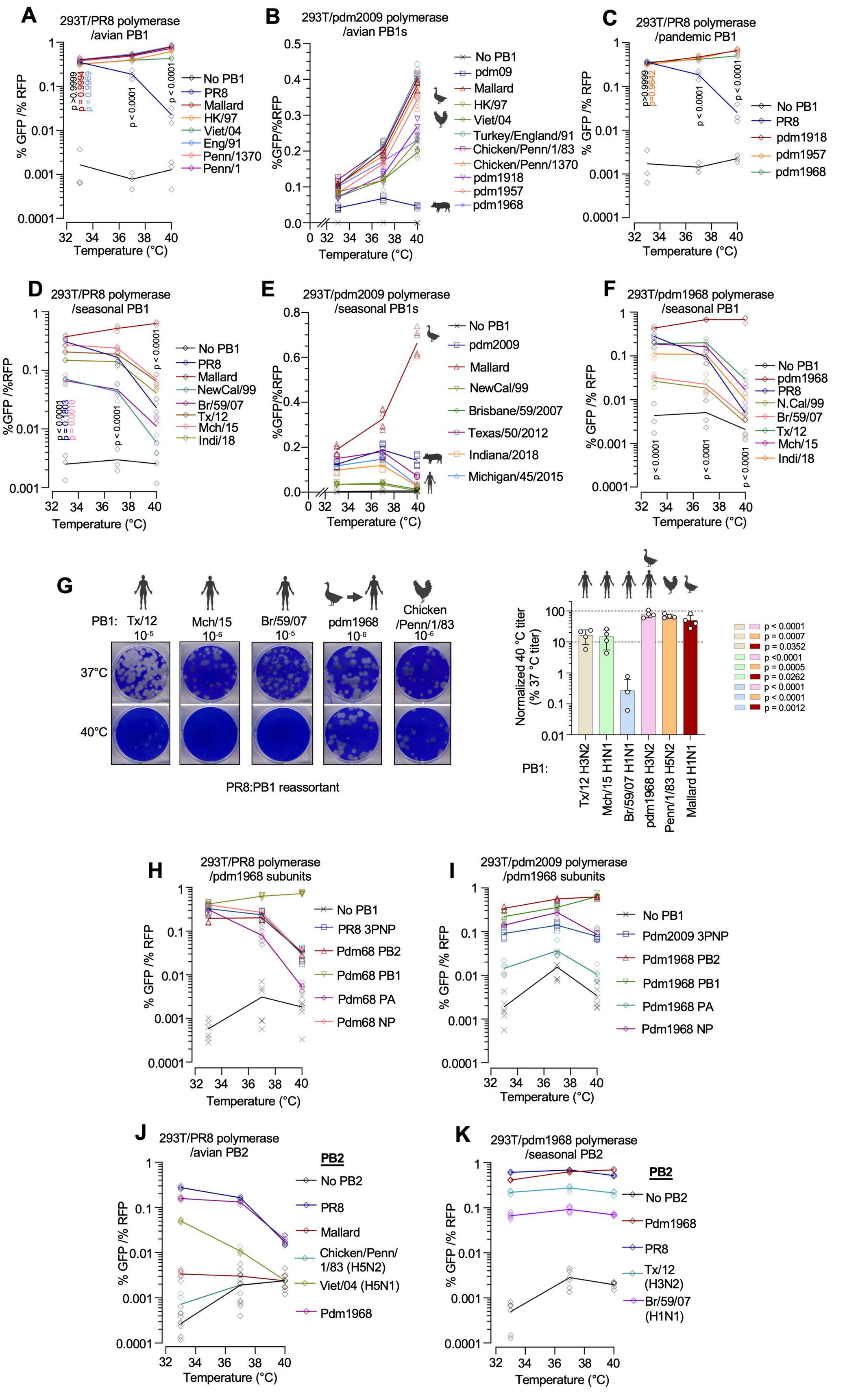
A
293T/PR8 polymerase
/avian PB1
% GFP /% RFP
Temperature (°C)
No PB1
PR8
Mallard
HK/97
Viet/04
Eng/91
Penn/1370
Penn/1
p < 0.0001
B
293T/pdm2009 polymerase
/avian PB1s
%GFP/%RFP
Temperature (°C)
No PB1
pdm09
Mallard
HK/97
Viet/04
Turkey/England/91
Chicken/Penn/1/83
Chicken/Penn/1370
pdm1918
pdm1957
pdm1968
C
293T/PR8 polymerase
/pandemic PB1
% GFP /% RFP
Temperature (°C)
No PB1
PR8
pdm1918
pdm1957
pdm1968
p < 0.0001
D
293T/PR8 polymerase
/seasonal PB1
%GFP /%RFP
Temperature (°C)
No PB1
PR8
Mallard
NewCal/99
Br/59/07
Tx/12
Mch/15
Indi/18
p < 0.0001
E
293T/pdm2009 polymerase
/seasonal PB1s
%GFP/%RFP
Temperature (°C)
No PB1
pdm2009
Mallard
NewCal/99
Brisbane/59/2007
Texas/50/2012
Indiana/2018
Michigan/45/2015
F
293T/pdm1968 polymerase
/seasonal PB1
% GFP /% RFP
Temperature (°C)
No PB1
pdm1968
PR8
N.Cal/99
Br/59/07
Tx/12
Mch/15
Indi/18
p < 0.0001
G
PB1:
Tx/12
Mch/15
Br/59/07
pdm1968
Chicken /Penn/1/83
37°C
40°C
PR8:PB1 reassortant
Normalized 40 °C titer (% 37 °C titer)
PB1:
Tx/12 H3N2
Mch/15 H1N1
Br/59/07 H1N1
pdm1968 H3N2
Penn/1/83 H5N2
Mallard H1N1
p < 0.0001
p = 0.0007
p = 0.0352
p <0.0001
p = 0.0005
p = 0.0262
p < 0.0001
p < 0.0001
p = 0.0012
H
293T/PR8 polymerase
/pdm1968 subunits
% GFP /% RFP
Temperature (°C)
No PB1
PR8 3PNP
Pdm68 PB2
Pdm68 PB1
Pdm68 PA
Pdm68 NP
I
293T/pdm2009 polymerase
/pdm1968 subunits
% GFP /% RFP
Temperature (°C)
No PB1
Pdm2009 3PNP
Pdm1968 PB2
Pdm1968 PB1
Pdm1968 PA
Pdm1968 NP
J
293T/PR8 polymerase
/avian PB2
% GFP /% RFP
Temperature (°C)
PB2
No PB2
PR8
Mallard
Chicken/Penn/ 1/83 (H5N2)
Viet/04 (H5N1)
Pdm1968
K
293T/pdm1968 polymerase
/seasonal PB2
% GFP /% RFP
Temperature (°C)
PB2
No PB2
Pdm1968
PR8
Tx/12 (H3N2)
Br/59/07 (H1N1)

Fig. S3. **Avian-origin PB1 enables temperature-resistant replication in mammalian cells**. **(A)** Polymerase activity from Fig. 2B is plotted without normalising to activity at 33˚C. (**B**) Polymerase activity from pandemic 2009 H1N1 polymerases with the indicated avian PB1 genes is plotted (as in A) Data are from two independent experiments performed in triplicate. (**C**) As in (A) for Fig 2C. (**D**) As in (A) for Fig 2D **(E)** Polymerase activity from pandemic 2009 H1N1 polymerases with the indicated seasonal PB1 genes is plotted (as in B). (**F**) As in (A) for Fig 2EStatistical significance was performed using a two-way ANOVA with Tukey's multiple comparisons test on data from three independent experiments (A, C, D and F) or individual data points from two independent experiments each performed in technical triplicate are plotted (B and E). (**G**) Infectious titer (plaque assay on MDCK cells) for the indicated PB1 reassortant virus (in a PR8 background) is plotted (as a percentage of the titer at 37˚C) from four independent experiments (Mean and SD). One data point (for Br/59/07) fell below the lowest y-axis value and is therefore not shown. P-values from an ordinary one-way ANOVA with Tukey's multiple comparisons comparing human and avian PB1 values are shown. (**H** and **I**) polymerase activity with the indicated pdm68 polymerase subunits is shown for PR8 (H) and pdm2009 (I) polymerases. (**J** and **K**) Polymerase activity with the indicated PB2 subunit is plotted for PR8 (J) and pdm1968 (K) polymerases. For H to K, individual data points are plotted from two independent experiments each performed in technical triplicate.

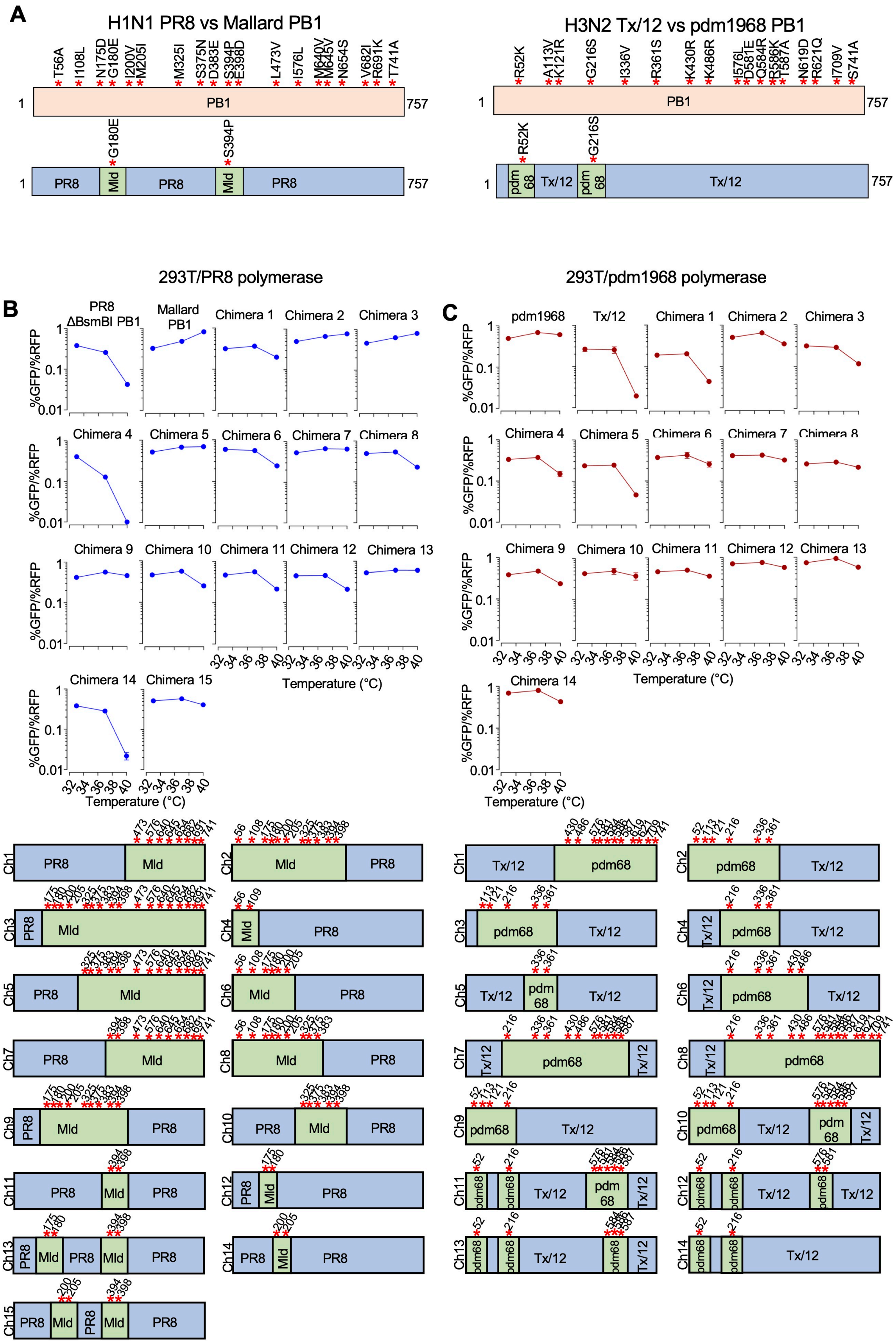
A
H1N1 PR8 vs Mallard PB1
T56A
I108L
N175D
G180E
I200V
M205I
M325I
S375N
D383E
S394P
E398D
L473V
I576L
M640V
M645V
N654S
V682I
R691K
T741A
PB1
1
757
G180E
S394P
PR8
Mld
H3N2 Tx/12 vs pdm1968 PB1
R52K
A113V
K121R
G216S
I336V
R361S
K430R
K486R
I576L
D581E
Q584R
R586K
T587A
N619D
R621Q
I709V
S741A
pdm 68
Tx/12
293T/PR8 polymerase
293T/pdm1968 polymerase
B
C
PR8 ΔBsmBI PB1
Mallard PB1
pdm1968
Chimera 1
Chimera 2
Chimera 3
Chimera 4
Chimera 5
Chimera 6
Chimera 7
Chimera 8
Chimera 9
Chimera 10
Chimera 11
Chimera 12
Chimera 13
Chimera 14
Chimera 15
%GFP/%RFP
Temperature (°C)
Ch1
Ch2
Ch3
Ch4
Ch5
Ch6
Ch7
Ch8
Ch9
Ch10
Ch11
Ch12
Ch13
Ch14
Ch15
pdm68

Fig. S4. Polymerase activity assay in 293T cells using chimeric PB1 proteins. (**A**) A simplified schematic of the amino acid changes in PB1 between H1N1 viruses PR8 and Mallard and the H3N2 viruses pdm68 and Tx/12 is shown (top). Numbering denotes the amino acid position of the PB1 protein. The dominant residues determined in polymerase assays and plaque reduction assays are shown (bottom). Mutations annotated are from PR8 to Mallard and Tx/12 to pdm68 residues, respectively. (**B** and **C**) Polymerase activity in the indicated polymerase background with the indicated PB1 is plotted (mean +/- SD) from three experimental replicates (top). A schematic representation of the PB1s used is also shown (bottom) highlighting which residues are derived from each strain. The PR8 chimera 4 polymerase had undetectable activity at 40°C and was therefore assigned a value of 0.01 (the lowest y-axis value).

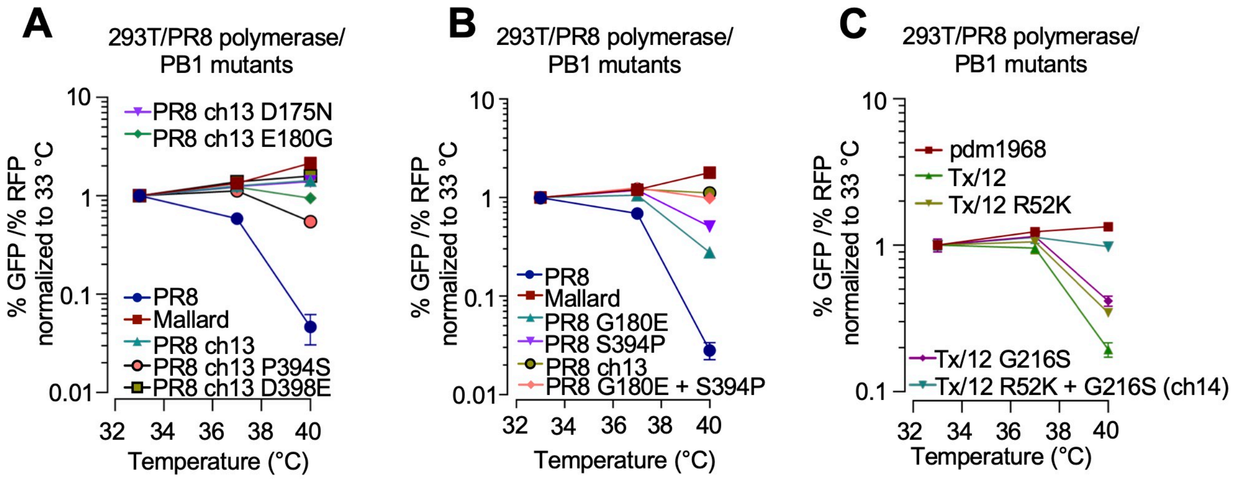

Fig. S5. Mapping of dominant temperature-resistant residues for the PR8: Mallard and Pdm1968: Tx/12 chimeric PB1 proteins. (**A** to **C**) Polymerase activity (PR8 background) with the indicated PB1 is plotted (mean +/- SD) from individual experiments performed in technical triplicate.

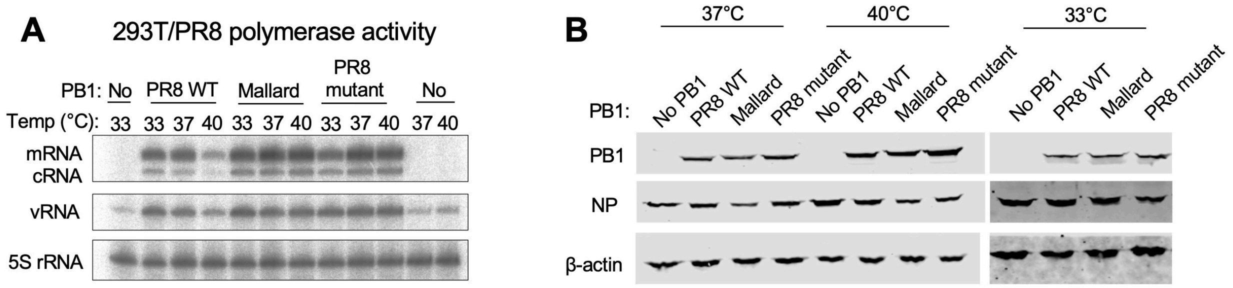

Fig. S6. Polymerase activity assay in human 293T cells. (A) Typical gel images (phosphorimager) underlying the data presented in Fig. 3D, visualizing the abundance of viral mRNA, cRNA and vRNA (^{32}P-labeled primer extension analysis) are shown. **(B)** Western blots visualizing PB1 and NP expression from cell lysates generated from RNA primer extension analyses in (A) and Fig. 3D.

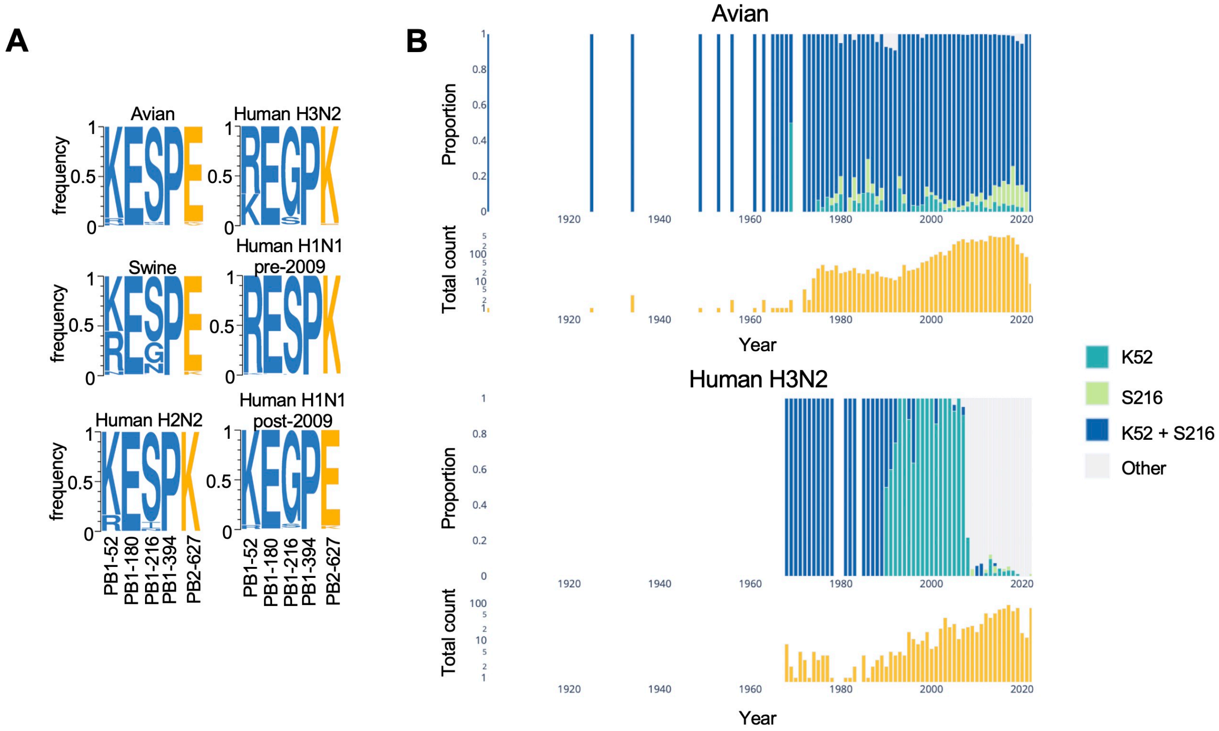

Fig. S7. **Distribution of PB1 residues**. (**A**) Web logos of the distribution of residues at PB1 sites 52, 180, 216 and 394 (blue) alongside PB2 avian-to-human-adaptation site 627 (yellow) are shown grouped by host species and virus lineage in humans. (**B**) The proportion of PB1 sequences containing K52, S216, both, or neither in circulating avian and human H3N2 IAVs is plotted over time.

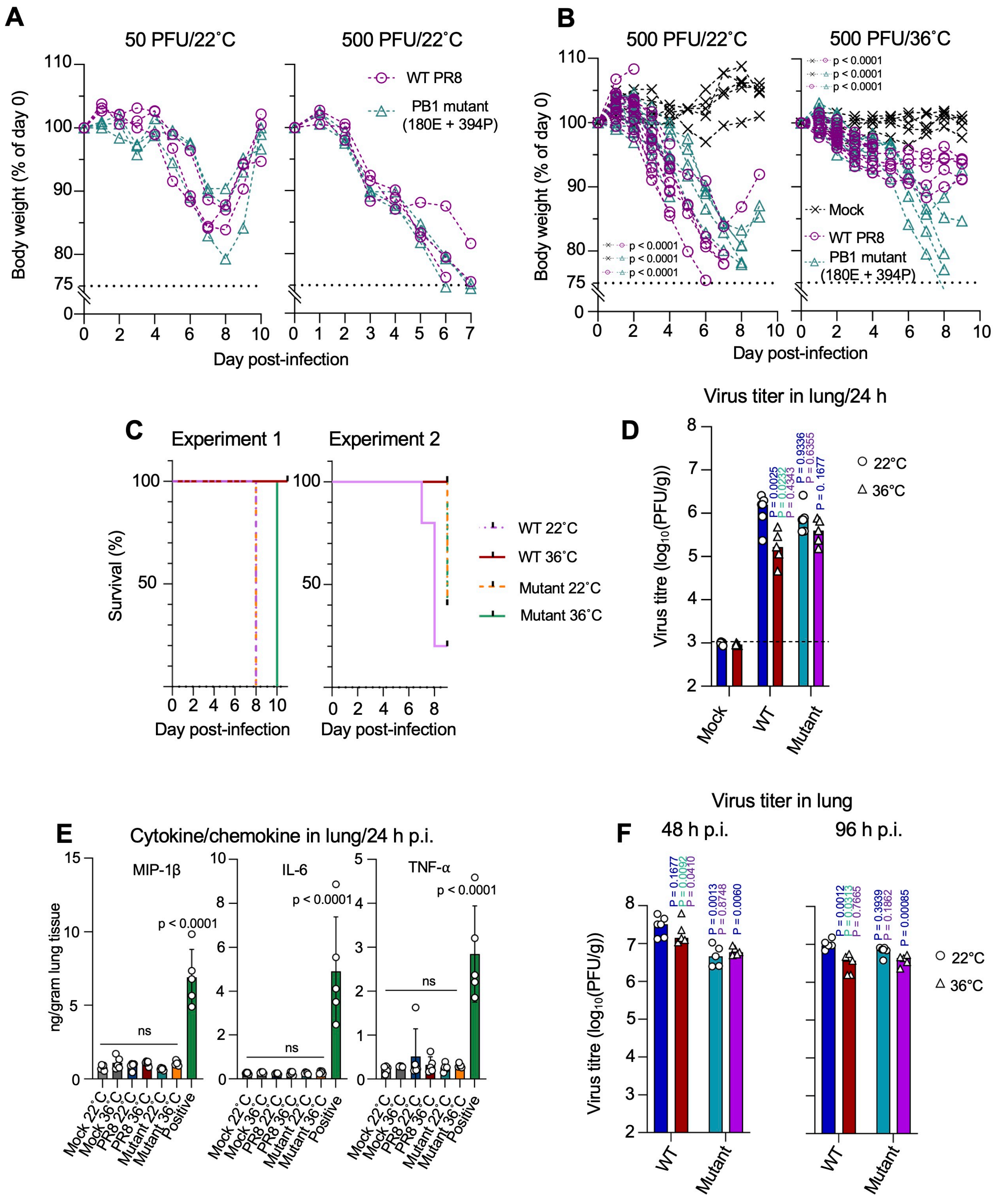

Fig. S8. ***In vivo* infections of WT and mutant PR8 viruses.** **(A)** Weight-loss curves from individual mice from groups of 3 C57BL/6 mice (intranasally inoculated with the stated dose of virus) housed at 22˚C. Horizontal dashed line indicates the ethical endpoint for euthanasia. (**B**) Weight-loss curves for individual mice from groups of 15 mice acclimatized to the indicated ambient temperature and intranasally infected or mock-infected are shown. 5 mice from each

group were euthanized at days 2 & 4 postinfection. The dashed line indicates the ethical endpoint. P-values were calculated using an ordinary one-way ANOVA with Tukey's multiple comparisons test on area under the curve analysis. (**C**) Kaplan-Meier survival plots for two independent experiments of a 500 PFU challenge of cohorts of 5 mice are shown (from the same experiments in Fig 3E and (B)). (**D**) Median infectious viral titer (plaque assay on MDCK cells) from lung homogenate (whole soft tissue of the lung) harvested 24 hours postinfection (500 PFUs) is plotted. Titers are the non-normalized titers displayed in Fig. 3G. (**E**) The levels of pro-inflammatory mediators in soft tissue of the lung (ELISA) from 24 hours postinfection (as in D) are plotted (mean +/- SD) from five mice in each group. Each value is a mean of an ELISA performed in technical duplicate. 'Positive' denotes a positive control cohort of mice infected with 500 PFU of PR8 at 48 h post-infection. P-values from an ordinary two-way ANOVA with main-effects only and Tukey's multiple comparisons test are shown (**F**) As in (D) harvested at 48 and 96 hours postinfection (from the mice in B). For D and F, p-values were calculated using two-way ANOVA analyses with Tukey's multiple comparisons on log-transformed data.

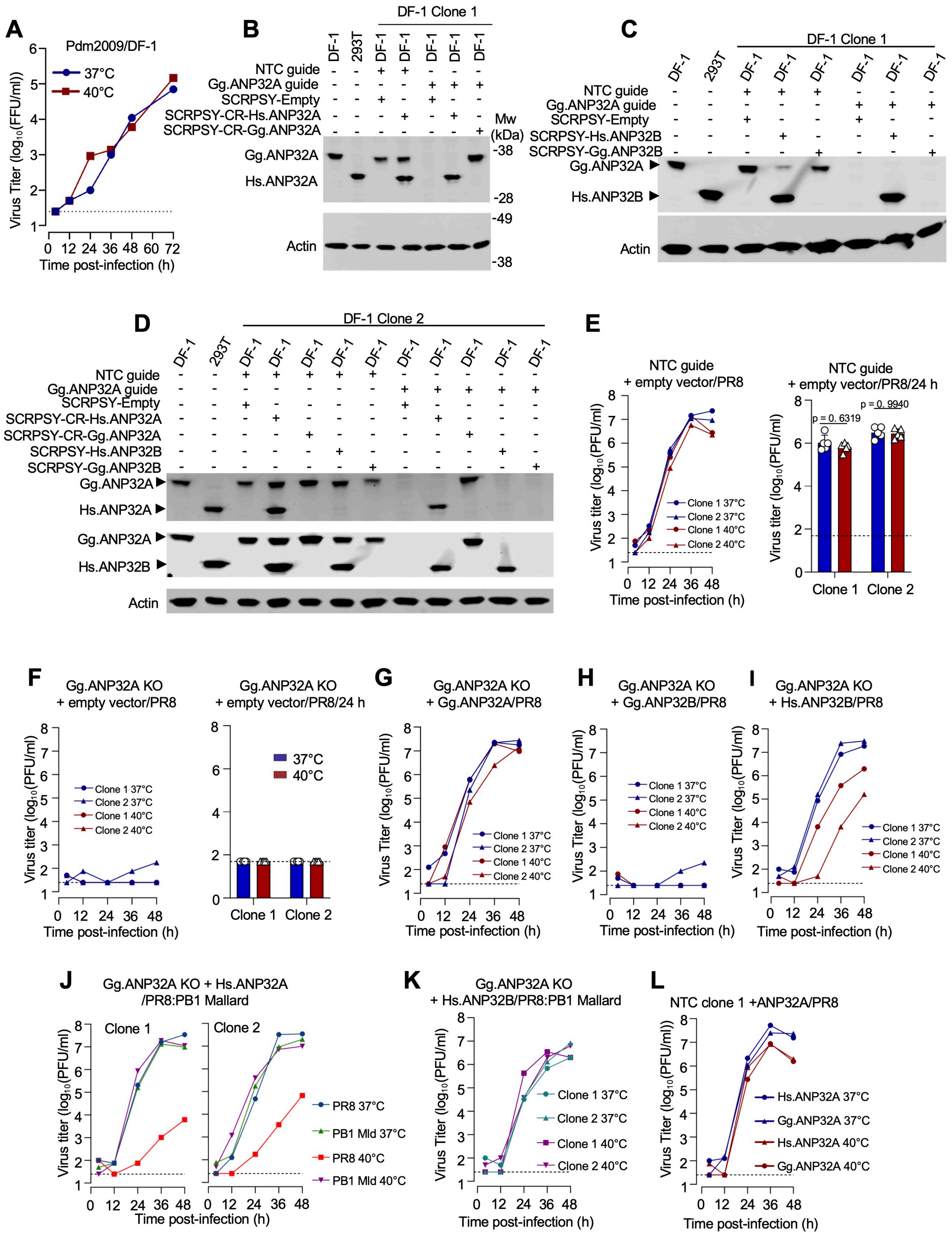
A
Pdm2009/DF-1
37°C
40°C
Virus Titer (log10(FFU/ml))
Time post-infection (h)
B
DF-1 Clone 1
NTC guide
Gg.ANP32A guide
SCRPSY-Empty
SCRPSY-CR-Hs.ANP32A
SCRPSY-CR-Gg.ANP32A
Mw (kDa)
Gg.ANP32A
Hs.ANP32A
Actin
C
DF-1 Clone 1
NTC guide
Gg.ANP32A guide
SCRPSY-Empty
SCRPSY-Hs.ANP32B
SCRPSY-Gg.ANP32B
Gg.ANP32A
Hs.ANP32B
Actin
D
DF-1 Clone 2
NTC guide
Gg.ANP32A guide
SCRPSY-Empty
SCRPSY-CR-Hs.ANP32A
SCRPSY-CR-Gg.ANP32A
SCRPSY-Hs.ANP32B
SCRPSY-Gg.ANP32B
Gg.ANP32A
Hs.ANP32A
Gg.ANP32A
Hs.ANP32B
Actin
E
NTC guide + empty vector/PR8
NTC guide + empty vector/PR8/24 h
Clone 1 37°C
Clone 2 37°C
Clone 1 40°C
Clone 2 40°C
p = 0. 6319
p = 0. 9940
Virus titer (log10(PFU/ml))
Time post-infection (h)
Clone 1
Clone 2
F
Gg.ANP32A KO + empty vector/PR8
Gg.ANP32A KO + empty vector/PR8/24 h
37°C
40°C
G
Gg.ANP32A KO + Gg.ANP32A/PR8
H
Gg.ANP32A KO + Gg.ANP32B/PR8
I
Gg.ANP32A KO + Hs.ANP32B/PR8
J
Gg.ANP32A KO + Hs.ANP32A /PR8:PB1 Mallard
Clone 1
Clone 2
PR8 37°C
PB1 Mld 37°C
PR8 40°C
PB1 Mld 40°C
K
Gg.ANP32A KO + Hs.ANP32B/PR8:PB1 Mallard
L
NTC clone 1 +ANP32A/PR8
Hs.ANP32A 37°C
Gg.ANP32A 37°C
Hs.ANP32A 40°C
Gg.ANP32A 40°C

Fig. S9. Chicken ANP32A can rescue temperature sensitive viral replication at an elevated temperature. (**A**) Infectious yield (focus forming units in MDCK cells) from DF-1 cells infected with pdm09 H1N1 virus (MOI 0.001 FFU/cell) is plotted. (**B**) Expression levels of chicken (Gg) and human (Hs) ANP32A were assessed in chicken DF-1 cell clones by western blotting using a primary antibody capable of detecting human and chicken ANP32A (top). Clones were modified using CRISPR-Cas9 with a nontargeting control guide (NTC) or a guide targeting chicken ANP32A. The indicated CRISPR-Resistant (CR) ANP32As were expressed using lentiviral transduction (SCRPSY). (**C**) Expression levels of chicken (Gg) and human (Hs) ANP32B were assessed using western blotting (as in B) using a primary antibody that detects Gg.ANP32A and Hs.ANP32A/B. (**D**) As in B and C, using independently generated clones (different KO guide RNA). The top panel was probed with an antibody specific for human and chicken ANP32A, while the middle panel was probed with an antibody that detects Gg.ANP32A as well as Hs.ANP32A/B. For panels B to D, the anti-actin controls were resolved using separate gels. (**E**) The infectious yield (plaque assay on MDCK cells) of PR8 (MOI of 0.001 PFU/cell) from DF-1 NTC clones over time (left) or from three independent experiments at 24 hours postinfection (right) is plotted. Dashed line indicates the limit of detection and p-values were calculated using two-way ANOVA with Tukey's multiple comparisons test on log transformed data. (**F**) The infectious yield of PR8 from ANP32A KO DF-1s is plotted as in E. (**G** to **I**) The infectious yield over time of PR8 from ANP32A KO DF-1 clones modified to express Gg.ANP32A (G), Gg.ANP32B (H) or Hs.ANP32B (I) using lentiviral transduction is plotted. Dashed line represents limit of detection.(**J**) Infectious yield over time of the indicated virus from ANP32A KO DF-1 cells modified to express Hs.ANP32A is plotted as in G. (**K**) The yield of PR8 with Mallard PB1 from ANP32A KO DF-1 clones modified to express Hs.ANP32B is plotted as in I. (**L**) The infectious yield of PR8 from DF-1 NTC clone 1 cells modified to express the indicated exogenous ANP32A protein over time is plotted as in G.

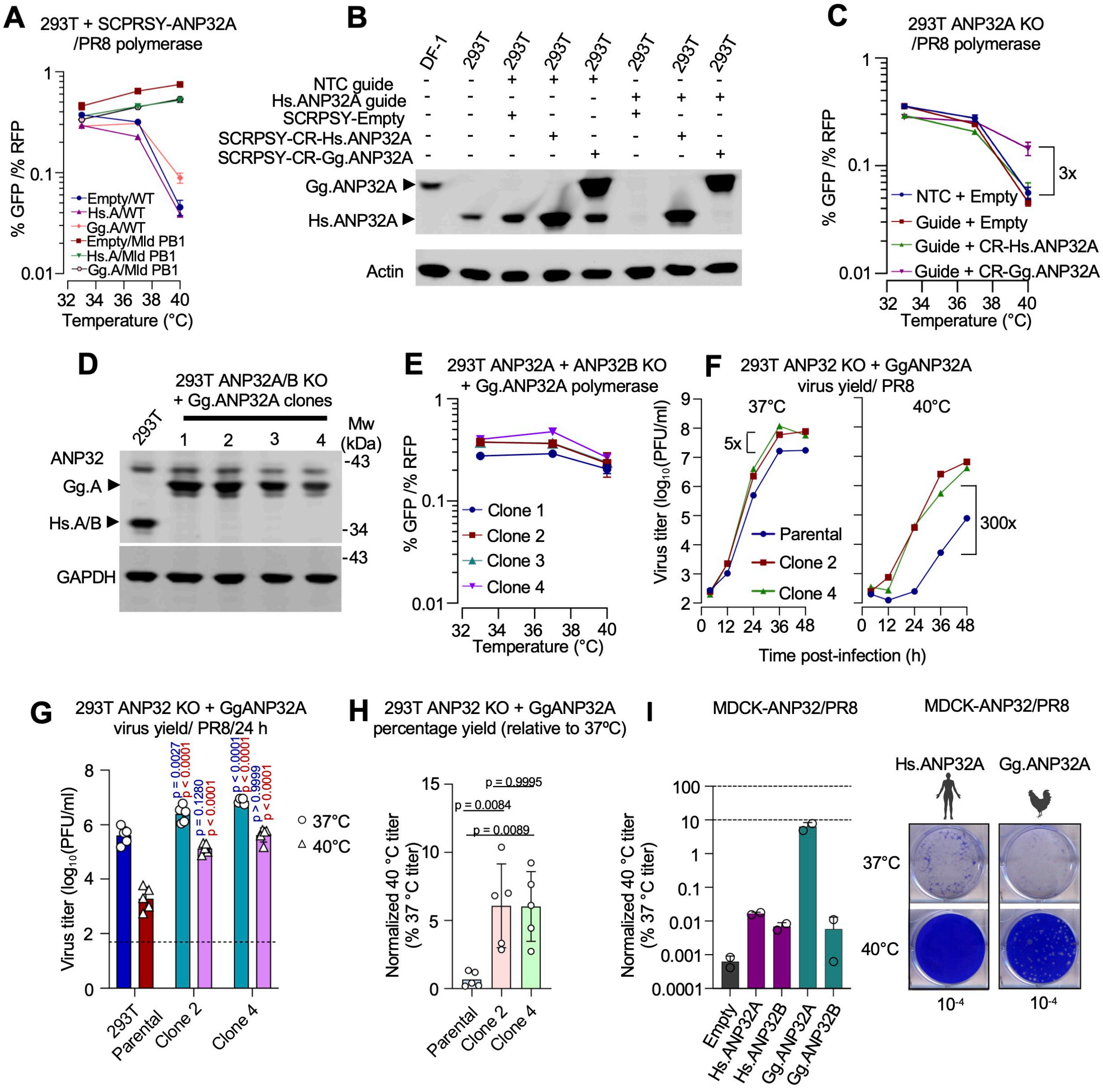

Fig. S10. Chicken ANP32A boosts temperature sensitive IAV replication in mammalian cells. (**A**) Polymerase activity (mean +/- SD) in transfected 293T cells modified to express human ANP32A (Hs.A) or chicken ANP32A (Gg.A) using lentiviral transduction are plotted using a PR8 polymerase background with PR8 PB1 (WT) or Mallard PB1. Assay performed in technical triplicate. (**B**) Expression levels of chicken (Gg) and human (Hs) ANP32A were assessed in human 293T cell clones by western blotting using a primary antibody capable of detecting human and chicken ANP32A and human ANP32A (top). Clones were modified using CRISPR-Cas9 with a nontargeting control guide (NTC) or a guide targeting human ANP32A. The indicated CRISPR-Resistant (CR) ANP32As were expressed using lentiviral transduction

(SCRPSY). (**C**) PR8 Polymerase activity is plotted as in A for the indicated transfected clone (from B). (**D**) ANP32 expression in four clones of ANP32A KO cells (from B) modified to express Gg.ANP32A (lentiviral transduction) prior to knockout of ANP32B was visualized using western blotting and an antibody recognizing Gg.ANP32A as well as Hs.ANP32A/B. (**E**) PR8 Polymerase activity is plotted as in A for the indicated transfected clone from D. (**F**) The infectious yield over time (plaque assay on MDCK cells) of PR8 (MOI of 0.001 PFU/cell) from the indicated clones (from B) is plotted at 37˚C (left) and 40˚C (right). Dashed line indicates the limit of detection. (**G**) The infectious yield (as in F) at 24 hours postinfection is plotted (mean +/- SD) and indicated p-values are from two-way ANOVA with Tukey's multiple comparisons test on log transformed data. Data are from five independent experiments. (**H**) The infectious yield (from G) is plotted as a percentage of the 37˚C titer and p-values were calculated using an ordinary one-way ANOVA with Tukey's multiple comparisons test. (**I**) Infectious titre of PR8 (plaque assay) is plotted (mean + SD) for two independent titrations on MDCK cells modified to express the indicated ANP32 protein (left) and example wells are shown (right).

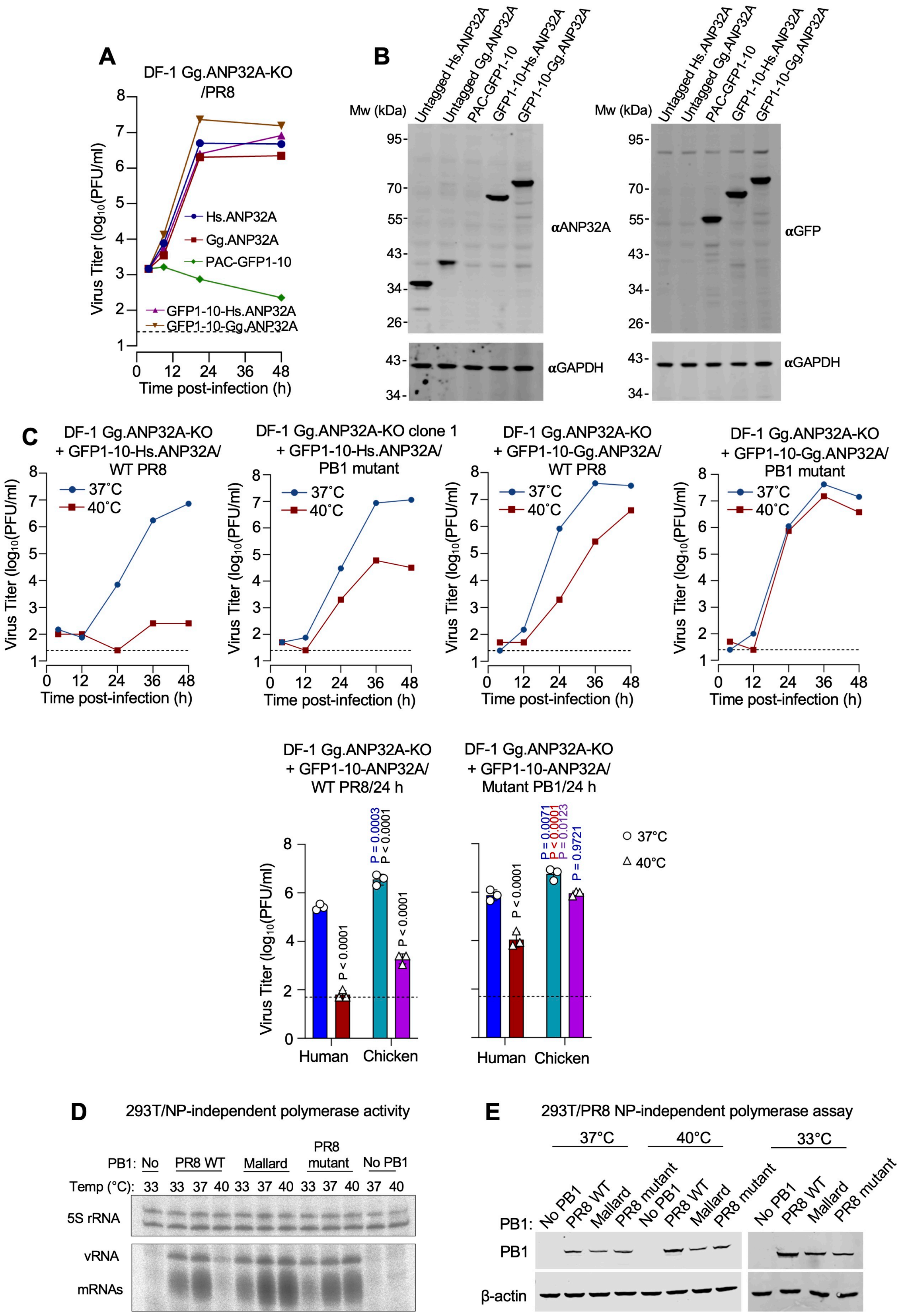
A
DF-1 Gg.ANP32A-KO /PR8
Virus Titer (log10(PFU/ml)
Time post-infection (h)
Hs.ANP32A
Gg.ANP32A
PAC-GFP1-10
GFP1-10-Hs.ANP32A
GFP1-10-Gg.ANP32A
B
Mw (kDa)
Untagged Hs.ANP32A
Untagged Gg.ANP32A
PAC-GFP1-10
GFP1-10-Hs.ANP32A
GFP1-10-Gg.ANP32A
αANP32A
αGFP
αGAPDH
C
DF-1 Gg.ANP32A-KO + GFP1-10-Hs.ANP32A/ WT PR8
DF-1 Gg.ANP32A-KO clone 1 + GFP1-10-Hs.ANP32A/ PB1 mutant
DF-1 Gg.ANP32A-KO + GFP1-10-Gg.ANP32A/ WT PR8
DF-1 Gg.ANP32A-KO + GFP1-10-Gg.ANP32A/ PB1 mutant
37˚C
40˚C
DF-1 Gg.ANP32A-KO + GFP1-10-ANP32A/ WT PR8/24 h
DF-1 Gg.ANP32A-KO + GFP1-10-ANP32A/ Mutant PB1/24 h
P = 0.0003
P < 0.0001
P = 0.0071
P < 0.0001
P = 0.0123
P = 0.9721
Human
Chicken
D
293T/NP-independent polymerase activity
PB1: No PR8 WT Mallard PR8 mutant No PB1
Temp (°C): 33 33 37 40 33 37 40 33 37 40 37 40
5S rRNA
vRNA
mRNAs
E
293T/PR8 NP-independent polymerase assay
37°C
40°C
33°C
PB1: No PB1 PR8 WT Mallard PR8 mutant
PB1
β-actin

Fig. S11. ANP32A-polymerase interaction is not broken at an elevated temperature. (**A**) The Infectious yield over time (plaque assay on MDCK cells) from DF1 ANP32A KO cells modified to express the indicated ANP32A and infected with PR8 (MOI of 0.01 PFU/cell) is plotted. GFP1-10 indicates the GFP tag used for the indicated ANP32As and puromycin N-acetyltransferase (PAC) control. Dashed line indicates the limit of detection. (**B**) ANP32 expression (left) and GFP expression (right) in the cells from A was visualized using western blotting. (**C**) Infectious yield over time of PR8 or the PB1 mutant (180E + 394P) in cells cultured at 37˚C or 40˚C is plotted as in A (top). Infectious yield at 24 hours (mean +/- SD) from three independent experiments is also plotted (below). Statistical significance was assessed by two-way ANOVA with Tukey's multiple comparisons on log-transformed data (**D**) Typical gel images (phosphorimager) underlying the data presented in Fig. 4E, visualizing the abundance of viral mRNA and vRNA (^{32}P-labeled NP-independent primer extension analysis) are shown. **(E)** Western blots visualizing PB1 expression from cell lysates generated from RNA primer extension analyses in (D) and Fig. 4E.

Table S1.

**Strain**	**Short name**	**PB2 GenBank accession**	**627 amino acid**
A/Puerto Rico/8/1934 (H1N1)	PR8	EF467818	K
A/mallard/Netherlands/10-Cam/1999 (H1N1)	Mallard or Mld	KC209512.1	E
A/Hong Kong/01/1968 (H3N2)	Pdm1968	KY321924.1	K
A/California/04-061-MA/2009 (H1N1)	Pdm2009	KX134889.1	E
A/Viet Nam/1194/2004 (H5N1)	Viet/04	AY651718.1	K
A/chicken/Pennsylvania/1/1983 (H5N2)	Penn/1/83	CY015080.1	E
A/Brisbane/59/2007 (H1N1)	Br/59/07	CY163871.1<br>With c1183t silent change to ablate BsmBI for cloning purposes.	K
A/Texas/50/2012 (H3N2)	Tx/12	KJ942623.1<br>With c1197t, c1842t, c2214t silent changes to ablate BsmBI sites for cloning purposes.	K

PB2 genes used in this study. For H5 avian strains, exclusively the PB2 ORF sequence, and not the UTR region essential to produce infective virus, was used. Where the UTR of the PB2 gene was incomplete in GenBank accessions for human seasonal viruses, the equivalent UTR from A/California/04/2009 (H1N1) (Genbank accession: MK159409.1) was used to form a complete genome segment.

Table S2.

Lineage	Residue	Reference AA count (R52/G180/G216/S394)	Alternate AA count (K52/E180/S216/P394)	Other AA count	No. of aligned sequences
1918 H1N1	R52K	711	50	2	763
1918 H1N1	G180E	15	747	1	763
1918 H1N1	G216S	97	661	5	763
1918 H1N1	S394P	3	759	1	763
1957 H2N2	R52K	8	44	0	52
1957 H2N2	G180E	0	52	0	52
1957 H2N2	G216S	0	48	4	52
1957 H2N2	S394P	0	52	0	52
1968 H3N2	R52K	2375	977	12	3364
1968 H3N2	G180E	4	3348	12	3364
1968 H3N2	G216S	3146	186	32	3364
1968 H3N2	S394P	2	3345	17	3364
2009 H1N1	R52K	43	2885	9	2937
2009 H1N1	G180E	1	2909	27	2937
2009 H1N1	G216S	2826	45	66	2937
2009 H1N1	S394P	0	2897	40	2937

PB1 amino acid frequency. Number of IAV PB1 sequences from each pandemic lineage containing residues identified in the study with a role in temperature sensitivity at sites 52, 180, 216 and 394. Lineage column refers to amino acid homology of PB1 to the representative PB1 sequence of the stated pandemic, and the virus strain may not necessarily share the same HA and NA subtype. Reference AA refers to R52, G180, G216 or S394, alternate refers to K52, E180, S216 or P394, and 'other' AA count refers to a different amino acid not tested in this study.

Table S3.

Strain	Host	Year	Subtype	TS phenotype	PB1-52	PB1-180	PB1-216	PB1-394
PR8	Laboratory-adapted	1934	H1N1	Sensitive	K	G	S	S
Br/59/07	Human	2007	H1N1 (pre-2009)	Sensitive	R	E	S	P
NewCal/99	Human	1999	H1N1 (pre-2009)	Sensitive	R	E	S	P
Indi/18	Human	2018	H3N2	Sensitive	R	E	G	P
Tx/12	Human	2012	H3N2	Sensitive	R	E	G	P
Mch/15	Human	2015	H1N1 (post-2009)	Sensitive	K	E	G	P
pdm1918	Avian > human	1918	H1N1	Resistant	K	E	S	P
pdm57	Avian > human	1957	H2N2	Resistant	K	E	S	P
pdm68	Avian > human	1968	H3N2	Resistant	K	E	S	P
Mallard	Mallard	1999	H1N1	Resistant	K	E	S	P
Penn/1/83	Chicken	1983	H5N2	Resistant	R	E	S	P
Penn/1370/83	Chicken	1983	H5N2	Resistant	R	E	S	P
HK/97	Avian>human	1997	H5N1	Resistant	K	E	S	P
Viet/04	Avian>human	2004	H5N1	Resistant	K	E	S	P
Turkey/Eng/91	turkey	1991	H5N1	Resistant	K	E	S	P

Summary of tested PB1s. PB1s tested in this study and the amino acid combination at sites 52, 180, 216 and 394. TS phenotype column is derived from *in vitro* polymerase activity assays described in Fig. 2 B to E and Fig S3A to F.

Table S4.

CRISPR-Cas9 guide	Clone number	ANP32 nucleotide edit (coding sequence numbering)	Corresponding disruption to protein coding	Estimated allelic frequency
ANP32A guide 1	1-4	Deletion ^{419}CGCAACT^{425}	Frameshift codons 141-182; introduces stop codon at codon 183	25%
		Deletion: ^{421}CAACTCA^{427}	Frameshift codons 141-182; introduces stop codon at codon 183	25%
		Deletion: ^{424}CT^{425}	Frameshift codons 142-147; introduces stop codon at codon 148	25%
		Insertion: ^{424}AATGTGTTCAAGCTCCTC^{441}	In-frame insertion of codons ^{142}N V F K L L^{147}	25%
ANP32B guide 1	2	Insertion: ^{447}C	Frameshift codons 150-156; stop codon introduced at codon 157	100%
ANP32B guide 2	4	Deletion: ^{64}CTTGT^{68}	Frameshift codons 23-28; stop codon introduced at codon 29	50%
		Deletion: ^{69}C	Frameshift codons 24-37; stop codon introduced at codon 37	50%

CRISPR-Cas9 genome edits of human ANP32A and ANP32B in 293T-Gg.ANP32A cells. Sites in ANP32A and ANP32B genes targeted for CRISPR-Cas9-editing in 293T clones were PCR amplified from genomic DNA using primers landing in flanking intronic regions to the guide targeting sites and the amplicon sequenced by Oxford Nanopore Technology. Analysis of these sequencing reads suggests the 293T cells were diploid for ANP32B and tetraploid for ANP32A, with the below edits noted and estimated allelic frequencies.

Table S5.

Strain	Short name	PB1 GenBank accession
A/Puerto Rico/8/1934 (H1N1)	PR8	EF467819
A/mallard/Netherlands/10-Cam/1999 (H1N1)	Mallard or Mld	KC209513.1
A/Hong Kong/01/1968 (H3N2)	Pdm1968	KY321925.1
A/California/04-061-MA/2009 (H1N1)	pdm2009	KX136823.1
A/Brevig Mission/1/1918 (H1N1)	pdm1918	Q3HM40.1
A/Singapore/1/1957 (H2N2)	Pdm1957	AAO46325.1
A/Hong Kong/481/97 (H5N1)	HK/97	AF115292.1
A/Viet Nam/1194/2004 (H5N1)	Viet/04	AY651664.1
A/turkey/England/50-92/1991 (H5N1)	Turkey/Eng/91	CY015125.1
A/chicken/Pennsylvania/1/1983 (H5N2)	Penn/1/83	CY015079.1
A/chicken/Pennsylvania/1370/1983 (H5N2)	Penn/1370/83	CY015113.1
A/Brisbane/59/2007 (H1N1)	Br/59/07	CY163870.1 with c1273a silent change to ablate *Bsm*BI site for cloning purposes
A/Texas/50/2012 (H3N2)	Tx/12	KJ942622.1
A/Michigan/45/2015 (H1N1)	Mch/15	KY117021.1
A/New Caledonia/20/1999 (H1N1)	NewCal/99	CY033628.1 with a552g and c1287g silent changes to ablate *Bsm*BI sites for cloning purposes
A/Indiana/08/2018 (H3N2)	Indi/18	MH306916.1

PB1 genes used in this study. Exclusively the PB1 ORF sequence, and not the UTR region essential to produce infective virus, was used for the pdm1918, pdm1957 and HPAI strains. Where the UTR of the PB1 gene was incomplete in GenBank accessions for human seasonal viruses, the equivalent UTR from A/California/7/2009 (H1N1) (Genbank accession: KC866601.1) was used to form a complete genome segment.

Table S6.

Chimera	Templates	Overlapping oligonucleotides
PR8/Mld ch1	PR8 PB1 (GenBank:EF467819)<br><br>Mallard PB1 (GenBank: KC209513.1)	5'-catcagaggattggagtccatcccaccagtaagtagtcttggtgtatctc-3'<br>5'- gagatacaccaagactacttactgg tgggatggactccaatcctctg-3'
PR8/Mld ch2	PR8 PB1 (GenBank:EF467819)<br><br>Mallard PB1 (GenBank: KC209513.1)	5'-cgtcagaggattgaagaccatcccaccagtatgtggttttggtgtacctc-3'<br>5'-gaggtacaccaaaaccacatactgg tgggatggtcttcaatcctctgac-3'
PR8/Mld ch3	PR8 PB1 (GenBank:EF467819)<br><br>Mallard PB1 (GenBank: KC209513.1)	5'-ccgattcattggctgtcagaccgtttgatctgaacacttctattgtgttgg-3'<br>5'-ccaacacaatagaagtgttcagatca aacggtctgacagccaatgaatcgg-3'
PR8/Mld ch4	PR8 PB1 (GenBank:EF467819)<br><br>Mallard PB1 (GenBank: KC209513.1)	5' cagactcattggccgtgaggccattcgatctgaaaacctctatagtgttgg-3'<br>5'-ccaacactatagaggttttcagatcg aatggcctcacggccaatgagtctg-3'
PR8/Mld ch5	PR8 PB1 (GenBank:EF467819)<br><br>Mallard PB1 (GenBank: KC209513.1)	5'- cattccctccaacggggagtccagattgttcaagtttctcacatatactcc-3'<br>5'-ggagtatatgtgagaaacttgaaca atctggactccccgttggagggaatg-3'
PR8/Mld ch6	PR8 PB1 (GenBank:EF467819)<br><br>Mallard PB1 (GenBank: KC209513.1)	5'-cattgcctccaactggcaaccctgattgctcaagtttctcacagatgctcc-3'<br>5'-ggagcatctgtgagaaacttgagca atcagggttgccagttggaggcaatg-3'
PR8/Mld ch7	PR8 PB1 (GenBank:EF467819)<br><br>Mallard PB1 (GenBank: KC209513.1)	5'-gctgtgccatctattagcagaggtcggatttttcaatcttctttcttgttg-3'<br><br>5'-caacaagaaagaagattgaaaaaatcc gacctctgctaatagatggcacagc-3'
PR8/Mld ch8	PR8 PB1 (GenBank:EF467819)<br><br>Mallard PB1 (GenBank: KC209513.1)	5'-gcagtcccctctattaagagcgatcttattttctcgattttctttctcgttg-3'<br>5'-caacgagaaagaaaatcgagaaaataa gatcgctcttaatagaggggactgc-3'
PR8/Mld ch9	PR8/Mld Chimera 2<br><br>PR8/Mld Chimera 3	5'-cgtcagaggattgaagaccatcccaccagtatgtggttttggtgtacctc-3'<br>5'-gaggtacaccaaaaccacatactgg tgggatggtcttcaatcctctgac-3'
PR8/Mld ch10	PR8/Mld Chimera 2<br><br>PR8/Mld Chimera 5	5'-cgtcagaggattgaagaccatcccaccagtatgtggttttggtgtacctc-3'<br>5'-gaggtacaccaaaaccacatactgg tgggatggtcttcaatcctctgac-3'
PR8/Mld ch11	PR8/Mld Chimera 2<br><br>PR8/Mld Chimera 7	5'-cgtcagaggattgaagaccatcccaccagtatgtggttttggtgtacctc-3'<br>5'-gaggtacaccaaaaccacatactgg tgggatggtcttcaatcctctgac-3'
PR8/Mld ch12	PR8/Mld Chimera 9<br><br>PR8 PB1 (GenBank:EF467819)	5'-gtgttatcattttcttagtcatattgtcccttactcttctctttctctgg-3'<br>5'-ccagagaaagagaagagtaagggacaatatgactaagaaaatgataacac-3'
PR8/Mld ch13	PR8/Mld Chimera 9<br><br>PR8/Mld Chimera 11	5'-gtgttatcattttcttagtcatattgtcccttactcttctctttctctgg-3'<br>5'-ccagagaaagagaagagtaagggacaatatgactaagaaaatgataacac-3'
PR8/Mld ch14	PR8/Mld Chimera 5<br><br>PR8/Mld Chimera 6	5'-gtgtgaccattttcttggtcatgttgtctctcacccgcctctttctctga-3'<br>5'-tcagagaaagaggcgggtgagagacaacatgaccaagaaaatggtcacac-3'
PR8/Mld ch15	PR8/Mld Chimera 5<br><br>PR8/Mld Chimera 11	5'-gtgtgaccattttcttggtcatgttgtctctcacccgcctctttctctga-3'<br>5'-tcagagaaagaggcgggtgagagacaacatgaccaagaaaatggtcacac-3'

Pdm1968Pdm1968/Tx/12 ch1	Pdm1968Pdm1968: KY321925.1<br><br>Tx/12: GenBank: KJ942622.1	5'-ccttattttctcaattttctttcttgttgattcattgaaatacttcagg-3'<br>5'-cctgaagtatttcaatgaatcaacaagaaagaaaattgagaaaataagg-3'
Pdm1968Pdm1968/Tx/12 ch2	Pdm1968: KY321925.1<br><br>Tx/12: GenBank: KJ942622.1	5'-ccttattttctcaattttcttccttgttgattcattgaaatactttagg-3'<br>5'-cctaaagtatttcaatgaatcaaca aggaagaaaattgagaaaataagg-3'
Pdm1968/Tx/12 ch3	Pdm1968/Tx/12 Chimera 2<br><br>Tx/12: GenBank: KJ942622.1	5' tgtgcatatccacttggctcattatcctcaggtagtggtccatcaattgg-3'<br>5'-ccaattgatggaccactacctgaggataatgagccaagtggatatgcaca-3'
Pdm1968/Tx/12 ch4	Pdm1968/Tx/12 Chimera 2<br><br>Tx/12: GenBank: KJ942622.1	5'-agaccattcgatctgaagacttctatggtgttggctaatgcagttgctgc-3'<br>5'-gcagcaactgcattagccaacaccatagaagtcttcagatcgaatggtct-3'
Pdm1968/Tx/12 ch5	Pdm1968/Tx/12 Chimera 2<br><br>Tx/12: GenBank: KJ942622.1	5'-cagaacgtttctgaaccattcaggttgattctttgtgatgtatgtaatcatc-3'<br>5'-gatgattacatacatcacaaagaatcaacctgaatggttcagaaacgttctg-3'
Pdm1968/Tx/12 ch6	Pdm1968/Tx/12 Chimera 8<br><br>Tx/12: GenBank: KJ942622.1	5'-caccgatatgtgtatctgtaatctttgatgaacagttgaagagccatctgg-3'<br>5'-ccagatggctcttcaactgttcatcaaagattacagatacacatatcggtg-3'
Pdm1968/Tx/12 ch7	Pdm1968/Tx/12 Chimera 8<br><br>Tx/12: GenBank: KJ942622.1	5'-cagacttcagggatgtgaagattccggatattgtataagtttggtcctcc-3'<br>5'-ggaggaccaaacttatacaatatcc ggaatcttcacatccctgaagtctg-3'
Pdm1968/Tx/12 ch8	Pdm1968: KY321925.1<br><br>Tx/12: GenBank: KJ942622.1	5' agaccattcgatctgaagacttctatggtgttggctaatgcagttgctgc-3'<br>5' gcagcaactgcattagccaacaccatagaagtcttcagatcgaatggtct-3'
Pdm1968/Tx/12 ch9	Pdm1968: KY321925.1<br><br>Tx/12: GenBank: KJ942622.1	5'-ccacttagtgttatccccagtgattgtgaaagaaagctctgtgtcttgtg-3'<br>5'-cacaagacacagagctttctttcacaatcactggggataacactaagtgg-3'
Pdm1968/Tx/12 ch10	Pdm1968/Tx/12 Chimera 7<br><br>Pdm1968/Tx/12 Chimera 9	5'-ccaatgctcatatcagctgactcatttattccagacactccaaaacttgg-3'<br>5'-ccaagttttggagtgtctggaataaatgagtcagctgatatgagcattgg-3'
Pdm1968/Tx/12 ch11	Pdm1968/Tx/12 Chimera 10<br><br>Tx/12: GenBank: KJ942622.1	5'-ggaagccgttcaacaaacaagggtggacaaactgacccaaggtcgtcagacc-3'<br>5'-cagtttgtccacccttgtttgttgaacggcttccatcgtttcaagacacgag-3'
Pdm1968/Tx/12 ch12	Pdm1968/Tx/12 Chimera 11<br><br>Tx/12: GenBank: KJ942622.1	5'-cgggtttggtcccacagcttctttatctcgaatgatctcctcgtc-3'<br>5'- cgaggagatcattcgagataaagaagctgtgggaccaaacccgctc-3'
Pdm1968/Tx/12 ch13	Pdm1968/Tx/12 Chimera 11<br><br>Tx/12: GenBank: KJ942622.1	5'-ctgtgggagcaaacccaatcaaggacaggactattggtttc-3'<br>5'- ctgaaaccaatagtcctgtccttgattgggtttgctcccac-3'
Pdm1968/Tx/12 ch14	Pdm1968/Tx/12 Chimera 11<br><br>Tx/12: GenBank: KJ942622.1	5'-ccacttagtgttatccccagtgattgtgaaagaaagctctgtgtcttgtg-3'<br>5'- cacaagacacagagctttctttcacaatcactggggataacactaagtgg-3'

Chimeric PB1 generation by overlap extension PCR. Shown are overlapping oligonucleotides and templates for each chimera. All overlap extension PCRs used the same terminal primers (forward: 5'-gcaacccgtctctggggagcgaaagcaggcaaaccatttg -3' and reverse: 5' -ggttgccgtctcttattagtagaaacaaggcattttttcatgaagg-3').
